# ClpP2 proteasomes and SpxA1 determine *Listeria monocytogenes* tartrolon B hyper-resistance

**DOI:** 10.1371/journal.pgen.1011621

**Published:** 2025-04-04

**Authors:** Tim Engelgeh, Sabrina Wamp, Patricia Rothe, Jennifer Herrmann, Martin A. Fischer, Rolf Müller, Sven Halbedel

**Affiliations:** 1 FG11 Division of Enteropathogenic bacteria and Legionella, Robert Koch Institute, Wernigerode, Germany; 2 Department of Microbial Natural Products, Helmholtz Centre for Infection Research and Department of Pharmaceutical Biotechnology, Helmholtz Institute for Pharmaceutical Research Saarland (HIPS) and Department of Pharmacy, Saarland University, Saarbrücken, Germany; 3 FG13 Division of Nosocomial Pathogens and Antibiotic Resistances, Robert Koch Institute, Wernigerode, Germany; 4 Institute for Medical Microbiology and Hospital Hygiene, Otto von Guericke University Magdeburg, Magdeburg, Germany; Texas A&M University, UNITED STATES OF AMERICA

## Abstract

The foodborne bacterium *Listeria monocytogenes* is transmitted to humans from various environmental sources through consumption of contaminated plant and animal-based food. *L. monocytogenes* uses ATP-binding cassette (ABC)-type drug transporters to resist antimicrobial compounds produced by competitors co-residing in its environmental reservoirs. We have shown previously that the TimAB transporter confers resistance of *L. monocytogenes* to tartrolon B, a boron containing macrodiolide produced by myxo- and proteobacterial species. Tartrolon B acts as a potassium ionophore and is sensed by TimR, the transcriptional repressor of *timABR* operon. We here have isolated tartrolon B resistant suppressor mutations outside the *timABR* locus. These mutations inactivated the *clpP2* gene, which encodes the main proteolytic component of house-keeping Clp proteases. Deletion of *clpP2* impaired growth and virulence but caused tartrolon B hyper-resistance. This phenotype was *timAB*-dependent, but neither production nor degradation of TimAB was affected upon *clpP2* inactivation. Combinatorial deletions of the genes encoding the three Clp ATPases showed that ClpCP2 and ClpXP2 proteasomes jointly promote tartrolon B hyper-resistance. Genetic follow-up experiments identified the ClpP2 substrate and transcription factor SpxA1 and its protease adaptor YjbH as further tartrolon B resistance determinants. SpxA1 activates transcription of the *cydABCD* operon encoding cytochrome oxidase and in accordance with this transposon mutants with impaired cytochrome oxidase function were depleted from a transposon mutant library during tartrolon B exposure. Our work demonstrates novel roles of Clp proteasomes, SpxA1 and cytochrome oxidase CydAB in the resistance against compounds dissipating transmembrane ion gradients and helps to better understand the genetic and chemical basis of the manifold ecological interactions of an important human pathogen in its natural ecologic niches.

## Author summary

*Listeria monocytogenes* is an environmental bacterium that can cause infections with a high mortality rate in humans if ingested together with contaminated food. In the environment, the bacterium is exposed to many antimicrobial compounds synthesized by competing microbial species. Tartrolons are a group of such compounds, as *L. monocytogenes* possesses an exporter highly specific for tartrolon B. We here investigated the mechanisms mediating tartrolon B resistance and found a second way of conferring tartrolon B resistance in addition to its transport out of the cell. We have elucidated this secondary mechanism and identified a network of ClpP2 proteasomes and the SpxA1 transcription factor, which is a known ClpP2 substrate, that jointly control resistance to tartrolon B. Tn-Seq experiments then showed that pathways ensuring cytochrome oxidase activity are specifically required for tartrolon B resistance. This explains the involvement of ClpP2 and SpxA1, as both regulate expression of genes encoding cytochrome oxidase subunits. Clp proteasomes and their substrates are crucial factors for the virulence of *L. monocytogenes*. Therefore, our characterisation of the ClpP2 proteasome network also provides new and essential insights into the basis of *L. monocytogenes* virulence.

## Introduction

Bacteria are constantly exposed to antimicrobial compounds synthesized by other species from surrounding microbiomes or by their plant or animal hosts to defend themselves against competitors and colonisation. *Listeria monocytogenes* is a Gram-positive bacterium that naturally occurs in the soil and in the gut of different animals and of humans. It is also frequently present on plant surfaces and found in surface waters as well as in coastal and estuarine water samples [1,2]. From these environmental resources, the bacterium is regularly introduced into food production facilities leading to food contamination [3]. Uptake of such contaminated food can lead to invasive listeriosis in immunosuppressed individuals such as pregnant women, elderly patients, patients with immunosuppressive comorbidities or a history of immunosuppressive therapy [1,4].

*L.* *monocytogenes* has developed manifold resistance mechanisms to withstand the exposure to antimicrobial compounds either produced by environmental competitors or its hosts. Compound extrusion by ATP binding cassette (ABC)-type drug transporters is seemingly a favoured way of resistance development, especially if the high number of ABC transporter genes present in the *L. monocytogenes* genome is taken into account [5,6].

*L. monocytogenes* has an ABC-type antimicrobial compound exporter that helps the bacterium to survive in the gastrointestinal tract by exporting bile salts [7], such ones that facilitate extrusion of disinfectants and selected cephalosporins [8,9] and others for the export of antimicrobial substances produced by different soil microorganisms [5,10]. The LieAB and TimAB exporters belong to the latter group of ABC-type drug transporters as they export aurantimycin and tartrolon B, respectively [5,10]. Both antibiotics are synthesized by soil-dwellers; aurantimycin by the actinobacterium *Streptomyces aurantiacus* [11] and tartrolon B by the myxobacterium *Sorangium cellulosum* [12,13]. Expression of the *lieAB* and *timAB* genes is repressed under standard cultivation conditions and induced only when *L. monocytogenes* comes in contact with aurantimycin and tartrolon B, respectively. This pattern of transcriptional regulation of the *lieAB* genes is maintained by the PadR-like repressor LftR in conjunction with its co-regulator LftS [10]. Remarkably, aurantimycin does not bind LftR or LftS directly *in vitro*, leaving the question as to how LftR senses aurantimycin unanswered [14]. In contrast, binding of TimR to the promoter of the *timABR* operon is relieved *in vitro* upon addition of tartrolon B, likely due to a direct interaction of tartrolon B and TimR [5]. As a consequence, the TimAB transporter is only produced upon contact with tartrolon B to mediate tartrolon B detoxification by excretion of the compound out of the cell [5].

Tartrolon B and its precursor tartrolon A are macrocyclic dilactones (macrodiolides) composed of two identical halves with 23 carbon atoms each. Four hydroxyl groups are oriented towards the centre of the macrocycle in tartrolon A. In tartrolon B, the oxygen atoms of these four hydroxyl groups are bound to a central boron atom [12,13]. Tartrolons A and B inhibit the growth of Gram-positive bacteria including *L. monocytogenes*, while Gram-negative bacteria are generally insensitive [5,13]. They act as potassium ionophores causing leakage of potassium out of the bacterial cell [5,13], which is toxic due to its detrimental effects on intracellular pH, turgor and membrane potential [15].

Homologs of the *timABR* gene cluster are found in *Listeria sensu stricto* species, such as *L. monocytogenes* and *L. innocua*, but are absent from the genomes of the related *Mesolisteria* species. Some of these *timABR*-negative *Mesolisteria* are hyper-resistant against tartrolon B, which suggests that additional mechanisms mediating tartrolon resistance must exist in the *Listeriaceae* [5].

To better understand the mechanisms of tartrolon action and compound detoxification, we have performed a screen for tartrolon B resistant suppressor mutants in *L. monocytogenes*. Mutations in the *timABR* locus conferred moderate tartrolon B resistance, while mutations conferring hyper-resistance mapped to the *clpP2* gene encoding the main proteolytic component of ATPase-dependent proteases [16,17]. Isolation of further suppressors and the analysis of their functional relationship uncovered a role of SpxA1 and its protease adaptor YjbH in tartrolon B resistance. Listerial SpxA1 is a redox-responsive transcriptional regulator that activates and represses a diverse set of genes important for anaerobic growth [18], but the protease network controlling SpxA1 stability remained undefined in *L. monocytogenes*. In *Bacillus subtilis*, Spx accumulates in *clpP* and *clpX* mutants [19] and degradation of *B. subtilis* Spx by ClpXP requires the adaptor protein YjbH [20]. *B. subtilis* Spx is also degraded by ClpCP complexed with the MecA adaptor *in vitro* [20], although Spx accumulation is not observed under standard growth conditions in the absence of *clpC* or *mecA* alone [19]. In contrast, ClpCP contributes to Spx turnover under disulfide stress, indicating that ClpXP is the primary and ClpCP a secondary protease for Spx degradation in *B. subtilis* [21]. Likewise, both ATPases must contribute to SpxA1 degradation in *L. monocytogenes*, as their simultaneous deletion results in the same tartrolon B hyper-resistance phenotype as observed with a Δ*clpP2* mutant, for which we demonstrate accumulation of SpxA1.

## Results

### Tartrolon B resistant suppressors with mutations in the *clpP2* gene

**Fig 1:**
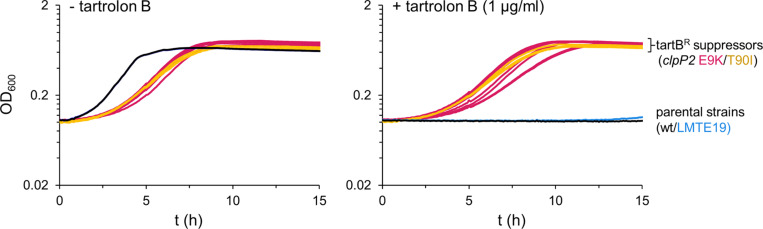
Tartrolon B resistant suppressors carry mutations in the *clpP2* gene. Growth of tartrolon B resistant suppressors (tartB^R^ suppressors) of strains LMTE19 as the parental strain, all carrying the *clpP2 E9K* mutation (LMTE38-40, LMTE42-49), and of EGD-e as the parent all carrying the *clpP2 T90I* mutation (LMTE74-76) in BHI broth at 37°C in the absence (left panel) or the presence of 1 µg/ml tartrolon B (right).

**Fig 2:**
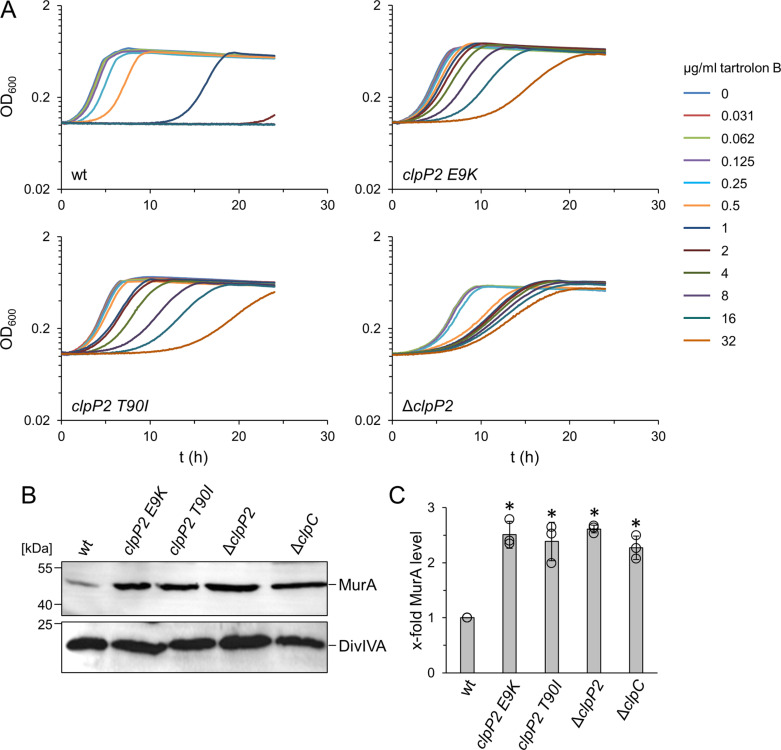
Effect of *clpP2* mutations on tartrolon B resistance and ClpP2 activity *in vivo.* (A) Growth of *L. monocytogenes strains* EGD-e (wt), LMTE38 (*clpP2 E9K*), LMTE74 (*clpP2 T90I*) and LMTE80 (Δ*clpP2*) in BHI broth containing increasing concentrations of tartrolon B at 37°C. (B-C) Western blot showing MurA (upper panel) and DivIVA levels (lower panel, loading control) in the same set of strains (B). A Δ*clpC* mutant (LMJR138) was included for comparison. MurA levels from three independent experiments were quantified by densitometry in ImageJ (C). Asterisks mark statistically significant differences (*P*<0.01, *t*-*t*est with Bonferroni-Holm correction).

To better understand regulation of tartrolon B resistance, we tried to isolate tartrolon B resistant suppressors. To this end, strain LMTE19 (a descendant of strain EGD-e, carrying the *attB::*P_*timABR*_*-lacZ* allele to monitor P_*timABR*_ promoter activity) [5] was inoculated in BHI broth containing 2 µg/ml tartrolon B (equals the minimal inhibitory concentration, MIC). Slight growth was observed after 42 hours and a dilution series was plated on BHI agar plates. Eleven clones were picked and streaked to single colonies. These clones grew slower compared to wild type when cultivated in BHI broth without tartrolon B, but could grow in the presence of 1 µg/ml tartrolon B, which was toxic for wild type under this condition (Fig 1). Genome sequencing showed that all eleven suppressors (LMTE38-LMTE40, LMTE42-LMTE49) had acquired a mutation in the *clpP2* gene (*lmo2468*) changing glutamate at position 9 into lysine (E9K) without the presence of any further mutations. The screen was repeated with EGD-e as the parental strain and again, suppressors were obtained that had a growth defect in plain BHI broth, but could withstand tartrolon B exposure in contrast to the wild type (Fig 1). Genome sequencing of three of these suppressors (LMTE74-LMTE76) identified a mutation in *clpP2* causing a T90I exchange together with a G241S substitution in the *lmo1296* gene, encoding a *hflXr* homologue without any apparent function [22], in all three strains. One suppressor was selected per type of *clpP2* mutation and growth was analyzed in the presence of increasing tartrolon B concentrations. This confirmed a higher MIC of tartrolon B for *clpP2 E9K* (LMTE38, >32 µg/ml) and *clpP2 T90I* strains (LMTE74, >32 µg/ml) than for the wild type (2 µg/ml Fig 2A). As no clear difference in growth and tartrolon B resistance was observed between the *clpP2 E9K* mutant and the *clpP2 T90I* mutant also carrying the substitution in *lmo1296*, we did not consider *lmo1296* further in this study. To confirm the role of *clpP2* in tartrolon B resistance, the *clpP2* gene was deleted in strain EGD-e. As expected, the Δ*clpP2* mutant could grow in the presence of the highest tartrolon B concentration tested in this experiment (>32 µg/ml, Fig 2A). In contrast, deletion of *clpP1* (*lmo1138*) did not affect tartrolon resistance (S1 Fig). Furthermore, resistance to boromycin, which is chemically related to the tartrolons and also acts as a potassium ionophore [23], but which is not transported by TimAB [5], was not affected in the Δ*clpP2* mutant (S2 Fig). This excludes the possibility that systems ensuring potassium homeostasis are deregulated in the absence of ClpP2 as an explanation for the high level tartrolon B resistance of the Δ*clpP2* mutant. Likewise, resistance to aurantimycin A, a chemically unrelated antimicrobial compound, but one which is also exported by an ABC-type MDR transporter is not increased (S2 Fig). Thus, the Δ*clpP2* mutant is not hyper-resistant against compounds exported by MDR transporters in general.

That the resistance against tartrolon B increased whether *clpP2* was deleted or mutated indicates that the E9K and T90I mutations inactivate or at least reduce the activity of ClpP2. To further test this idea, we analyzed intracellular levels of MurA, a known ClpCP substrate in *L. monocytogenes* [17,24–26], in the different *clpP2* mutant strains by Western blotting. This demonstrated that MurA accumulates in *clpP2 E9K* and *T90I* mutants to the same extent as seen in mutants that lack the *clpP2* gene or the *clpC* gene, encoding the corresponding ATPase (Figs 2B, 2C and S3). This shows that E9K and T90I exchanges strongly reduce ClpP2 activity.

### Effect of *clpP2* mutations on growth and virulence

Previously published work reported that deletion of *clpP2* did not result in a detectable growth defect despite the requirement of ClpP2 for protein turnover of many house-keeping proteins [17]. In contrast to this report, we observed that the *clpP2* mutant generated here forms smaller colonies than the wild type on BHI agar plates. We sequenced the genome of the Δ*clpP2* strain LMTE80, which confirmed the absence of *clpP2* specific reads and unwanted second site suppressor mutations. Further analysis showed that the Δ*clpP2* mutant in fact had a strong growth defect when grown in BHI broth at 37°C and could not grow at all at 42°C (S4A Fig). Less pronounced but concordant growth defects were also observed for *clpP2 E9K* and *T90I* strains (S4A Fig). Importantly, the inability of the *clpP2* mutant to grow at 42°C was partially complemented by reintroduction of *clpP2* (S4B Fig). Heat sensitivity explains the categorization of *clpP2* as an essential gene in our recent Tn-Seq study [27].

Next, we wondered how deletion/mutation of *clpP2* would impact intracellular growth. For this, J774 mouse macrophages were infected with wild type and *clpP2* mutants and samples were taken 6 hours post infection. This showed that deletion of *clpP2* decelerated intracellular growth substantially and that intracellular replication of the *clpP2 E9K* and *T90I* mutants was also retarded (S4C Fig). Taken together, we confirm that *clpP2* is required for growth of *L. monocytogenes*, particularly at higher temperature, and also that *clpP2* contributes to virulence, as was reported by other researchers [17,28].

### Tartrolon B-resistant suppressors in *tim* genes

**Fig 3:**
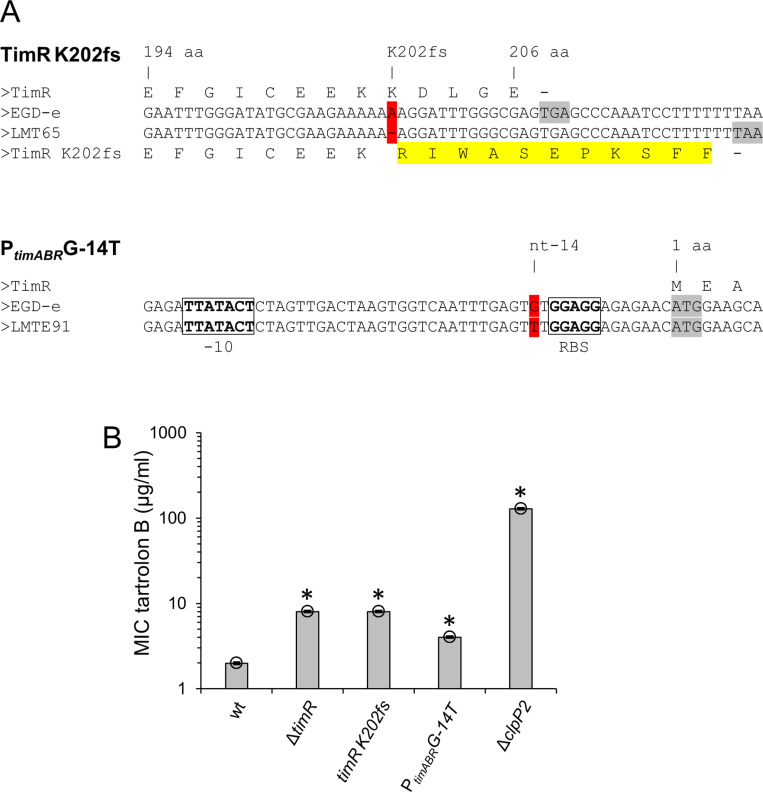
Effect of *timR* and P_*timABR*_ suppressor mutations on *L. monocytogenes* tartrolon B resistance. (A) Suppressor mutations in *timR* (strain LMTE65) and the P_*timABR*_ promoter (strain LMTE91) conferring intermediate levels of tartrolon B resistance. Mutated residues are highlighted in red, start and stop codons in grey and the frameshifted protein sequence is shown in yellow. Promoter elements are marked by boxes. (B) Minimial inhibitory tartrolon B concentrations of the suppressor strains shown in panel A. Strains LME37 (Δ*timR*) and LMTE80 (Δ*clpP2*) were included for comparison. Average values and standard deviations were calculated from three repetitions. Asterisks mark statistically significant differences compared to wild type. (*P*<0.01, *t*-*t*est with Bonferroni-Holm correction).

The vast majority of the tartrolon-resistant suppressors formed small colonies likely carrying *clpP2* inactivating mutations. However, a single suppressor with normal colony size was also obtained with EGD-e as the parental strain. Genome sequencing showed that the 604^th^ nucleotide of *timR* was deleted in this suppressor (LMTE65). The frameshift caused by this deletion (K202fs) resulted in a 212 amino acid long TimR variant (wild type length: 206 aa) with a non-natural protein sequence beyond its 201^st^ amino acid codon (Fig 3A) and in the same intermediate resistance level as a Δ*timR* mutant (MIC: 8 µg/ml, Fig 3B). To further force the appearance of suppressor mutations outside *clpP2*, we repeated the screen for tartrolon B-resistant suppressors with a strain that carried a second copy of the *clpP2* gene (LMPR7) and seven tartrolon B-resistant suppressors were isolated. Genome sequencing of three of them showed that all of them carried the same mutation in the P_*timABR*_ promoter (Fig 3A), enhancing tartrolon B resistance two-fold (4±0 µg/ml) compared to wild type (2±0 µg/ml, Fig 3B) presumably by increasing TimAB production. The resistance levels of *tim* suppressors differed from the Δ*clpP2* mutant, which reached the remarkable level 128±0 µg/ml, when a longer concentration gradient than before was used (Fig 3B). That *tim* mutations are directly selected when the possibility to escape tartrolon selection through *clpP2* mutations is excluded, suggests, that with *timABR* and *clpP2*, the elements of the main pathways controlling tartrolon resistance have been found. That *clpP2* deletion confers tartrolon B hyper-resistance is a second important conclusion.

### Distinct modes of action for tartrolon B and ADEP2

**Fig 4:**
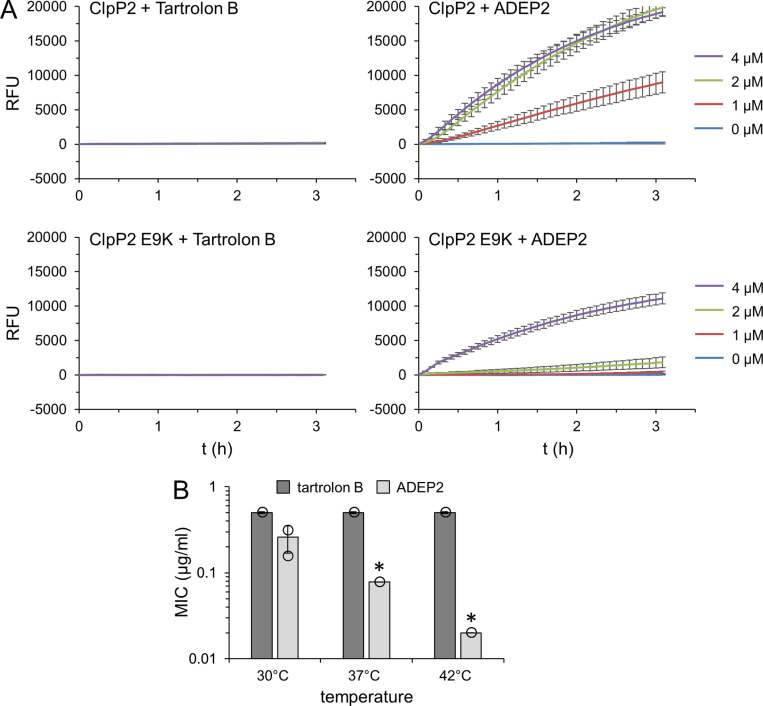
Effect of tartrolon B on ClpP2 activity *in vitro* and *in vivo.* (A) Effect of tartrolon B (left panels) and ADEP2 (right panels, for control) on ClpP2 and ClpP2 E9K peptidase activity *in vitro* using N-Succinyl-Leu-Tyr-7-amido-4-methylcoumarin as model substrate. Fluorescence was measured and corrected for background values. Assays were performed in triplicate and average values and standard deviations are shown. RFU – relative fluorescence units. (B) Effect of various growth temperatures on the susceptibilities of *L. monocytogenes* strain EGD-e against tartrolon B and ADEP2. The experiment was repeated three times and average values and standard variations are shown. Asterisks mark statistically significant differences (*P*<0.05, *t*-*t*est with Bonferroni-Holm correction).

We reasoned that tartrolon B could activate ClpP2 and that the E9K/T90I mutations compensate this by reducing ClpP2 activity. Activation of ClpP by natural compounds is not unprecedented as ADEP, an acyldepsipeptide produced by streptococci, is a known activator of ClpP2 proteins from different species [29,30]. To test this, we purified *L. monocytogenes* ClpP2-Strep (S5 Fig) and tested activation of its peptidase activity through ADEP2 and tartrolon B in an *in vitro* assay with Suc-Leu-Tyr-7-amido-4-methylcoumarin as the substrate. This showed that tartrolon B did not activate ClpP2, whereas a strong activation was seen with ADEP2 (Fig 4A). Purified ClpP2 E9K-Strep (S5 Fig) also did not respond to the presence of tartrolon B, even though its peptidase activity could still be activated by ADEP2 (Fig 4A). ADEP-dependent ClpP activation comes along with inhibition of ClpP-dependent protein degradation *in vivo* [30]. To further falsify the hypothesis that considered tartrolon B as an inhibitor of ClpP2-dependent protein degradation, we compared the susceptibilities of *L. monocytogenes* wild type strain EGD-e against tartrolon B and ADEP2 at different temperatures. We reasoned that the susceptibility against a ClpP2 inhibitor would increase with increasing temperature as *clpP2* becomes essential particularly at higher temperatures (as shown above). In good agreement with this assumption, ADEP susceptibility increased ~10-fold when the temperature was raised from 30°C to 42°C, however, tartrolon B resistance was clearly temperature-independent (Fig 4B). This shows that tartrolon B must act on another target than ClpP2, a finding that is concordant with the previously reported conclusion that tartrolon B is a potassium ionophore [5].

### Tartolon hyper-resistance of the *clpP2* mutant is TimAB-dependent

**Fig 5:**
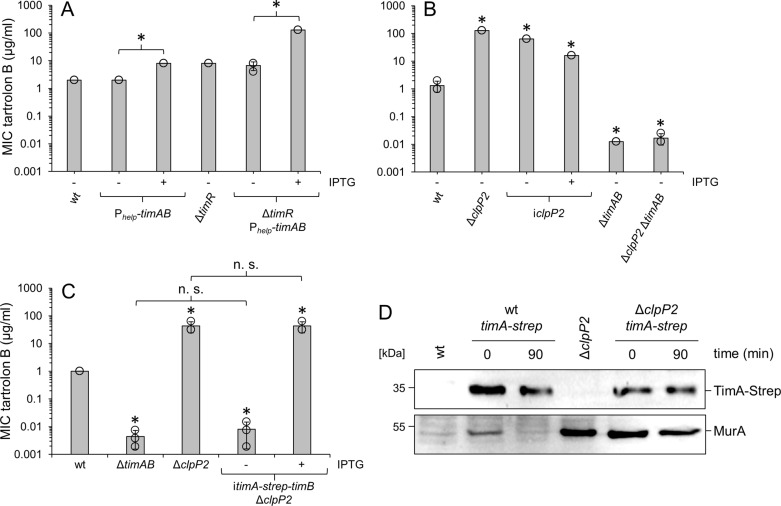
Tartrolon B resistance of the Δ*clpP2* mutant is *timAB*-dependent but TimA is not a substrate of ClpP2. (A) Minimal inhibitory tartrolon B concentrations after *timAB* overexpression in *L. monocytogenes*. Strains used were EGD-e (wt), LMTE89 (P_*help*_*-timAB*), LMTE37 (Δ*timR*), LMTE94 (Δ*timR* P_*help*_*-timAB*). (B) TimAB dependence of the tartrolon B hyper-resistance observed in the Δ*clpP2* mutant. Minimal inhibitory tartrolon B concentrations for *L. monocytogenes* strains EGD-e (wt), LMTE80 (Δ*clpP2*), LMTE90 (i*clpP2*), LMTE34 (Δ*timAB*) and LMTE95 (Δ*clpP2* Δ*timAB*). (C) TimA-Strep has complementation activity. Minimal inhibitory concentrations of tartrolon B for *L. monocytogenes* strain LMTE110 (i*timA-strep-timB* Δ*clpP2*) under non-inducing (-IPTG) and inducing conditions (+IPTG). Strains EGD-e (wt), LMTE34 (Δ*timAB*) and LMTE80 (Δ*clpP2*) were included as controls. In panels A-C, average values and standard deviations, which were calculated from three independent repetitions, are shown. Asterisks mark statistical differences compared to wild type (*P*<0.05, *t*-*t*est with Bonferoni-Holm correction). n. s. – not significant. (D) TimA is not a ClpP2 substrate. Western blots showing TimA-Strep (top panel) and MurA levels (bottom panel) in i*timA-strep-timB* strains LMT106 (labelled “wt *timA-strep*”) and LMTE110 (labelled “Δ*clpP2 timA-strep*”). Strains were grown in BHI broth (containing IPTG) at 37°C to an OD_600_ of 1.0 and protein biosynthesis was blocked by addition of chloramphenicol. Samples were taken before and 90 min after chloramphenicol addition. Strains EGD-e (wt) and LMTE80 (Δ*clpP2*) were included as controls.

We noticed that artificial overexpression of *timAB* was sufficient to further increase tartrolon B resistance in wild type background (Fig 5A). Likewise, *timAB* overexpression further enhanced tartrolon B resistance in the Δ*timR* background to the remarkable level of 128±0 µg/ml, which equals the resistance level of the Δ*clpP2* mutant (Fig 5A and 5B). Importantly, reintroduction of an IPTG-controlled *clpP2* copy into the Δ*clpP2* mutant complemented this phenotype in an IPTG-dependent manner (without IPTG: 64±0 µg/ml; with IPTG: 16±0 µg/ml, *P*≤0.01; Fig 5B). We also constructed a Δ*clpP2* Δ*timAB* mutant and determined its susceptibility against tartrolon B. Surprisingly, the Δ*clpP2* Δ*timAB* mutant (0.017±0 µg/ml) was as sensitive to tartrolon B as the Δ*timAB* single mutant (0.0125±0 µg/ml, *P*>0.05), indicating that the high tartrolon B resistance of the Δ*clpP2* mutant could involve *timAB*.

Tartrolon B hyper-resistance of the *clpP2* mutant could result from increased TimAB levels, caused by reduced TimAB degradation, provided that TimA or TimB would be ClpP2 substrates. We reasoned that this is unlikely for the membrane-embedded TimB. To test whether TimA could be a Clp substrate, we constructed a Δ*timAB* strain that expresses an IPTG-inducible *timA-strep-timB* allele (with TimA carrying a C-terminal Strep-Tag, strain LMTE106), allowing for TimA detection by Western blotting using an anti-Strep antibody, and then removed *clpP2* from this background (LMTE110). This latter strain showed IPTG-dependent tartrolon resistance spanning the entire range of observed resistance levels, from the low level of the Δ*timAB* mutant (when grown in the absence of IPTG) up to the high level of the Δ*clpP2* mutant (when cultivated with IPTG), demonstrating functionality of TimA-Strep (Fig 5C). However, in stark contrast to the ClpP2 substrate MurA, TimA-Strep levels were similar in wild type and Δ*clpP2* strains. Moreover, TimA-Strep was stable for 90 minutes after blocking protein biosynthesis by addition of chloramphenicol in wild type and Δ*clpP2* strains, whereas MurA was only stable in the absence of *clpP2* (Figs 5D and S3). Both observations speak against the idea that TimA is a substrate of ClpP2.

### Contribution of Clp ATPases to tartrolon B hyper-resistance

**Fig 6:**
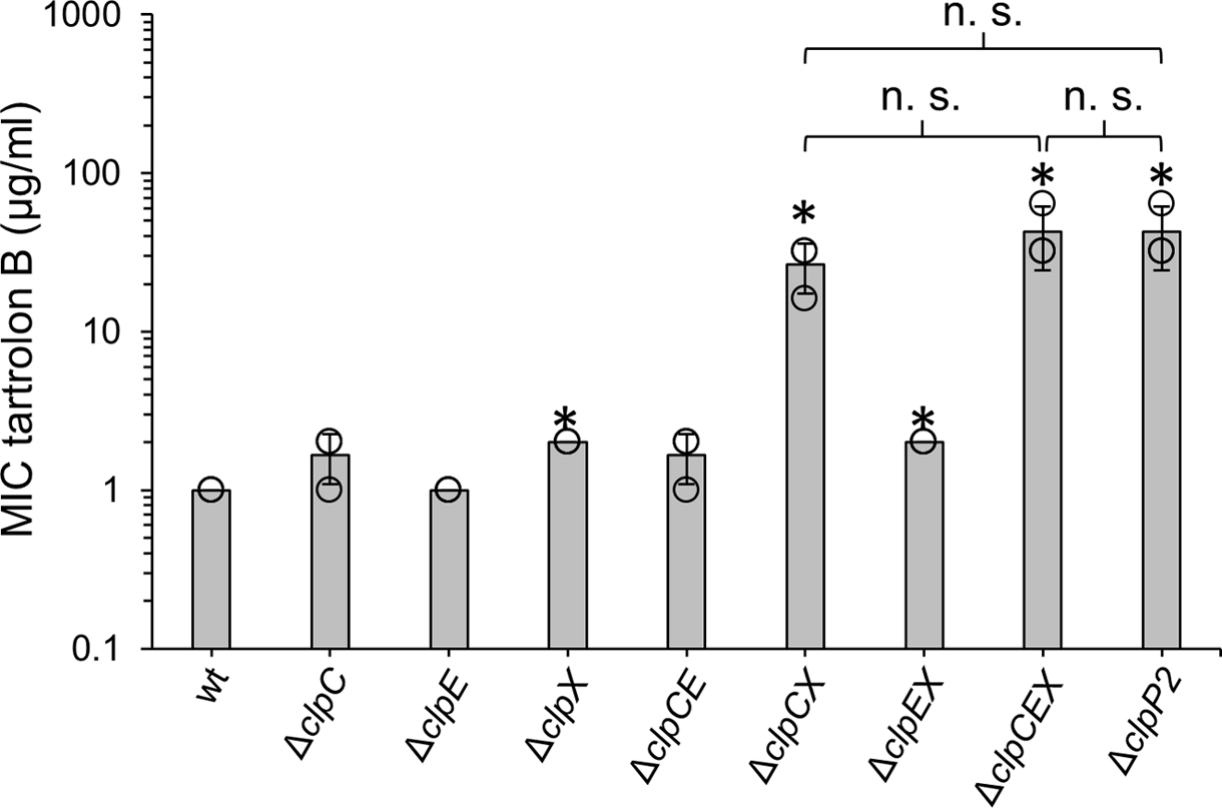
Role of Clp ATPases in tartrolon B resistance. Minimal inhibitory tartrolon B concentrations for *L. monocytogenes* strains EGD-e (wt), LMJR138 (Δ*clpC*), LMTE100 (Δ*clpE*), LMTE113 (Δ*clpX*), LMTE102 (Δ*clpE* Δ*clpC*), LMTE112 (Δ*clpE* Δ*clpX*), LMTE114 (Δ*clpC* Δ*clpX*), LMTE115 (Δ*clpC* Δ*clpE* Δ*clpX*) and LMTE80 (Δ*clpP2*). The experiment was repeated three times and average values and standard deviations are shown. Asterisks mark statistical significance (*P*<0.05, *t*-*t*est). n. s. – not significant.

ClpP proteases associate with AAA+ ATPases to form functional proteasomes, where the ATPases catalyze ATP-dependent unfolding of substrate proteins and their translocation to ClpP for degradation [31]. *L. monocytogenes* contains three Clp ATPases, *i.e.,* ClpC, ClpE and ClpX [32–34] assembling into distinct proteasomes with ClpP2 and each of them is considered to degrade specific sets of substrates. In order to test, which of the three ATPases contributes to tartrolon B resistance, we constructed mutants lacking *clpC*, *clpE* or *clpX* and determined their tartrolon B MICs. A twofold (and significant) increase of the MIC was observed for the *clpX* mutant, while deletion of *clpC* or *clpE* had no significant effect (Fig 6). Likewise, a Δ*clpCE* double mutant showed wildtype-like resistance, and a Δ*clpEX* double mutant was as resistant as a Δ*clpX* single mutant strain (Fig 6). However, when Δ*clpC* and Δ*clpX* deletions were combined in one strain, then a Δ*clpP2*-like increase of the tartrolon B MIC was obtained, and the same Δ*clpP2*-like resistance level was observed in a Δ*clpCEX* triple mutant (Fig 6). This shows that the hyper-resistance of the Δ*clpP2* mutant against tartrolon B involves ClpC and ClpX, while ClpE is not important.

### Tartrolon B resistance depends on YjbH and SpxA1

**Fig 7:**
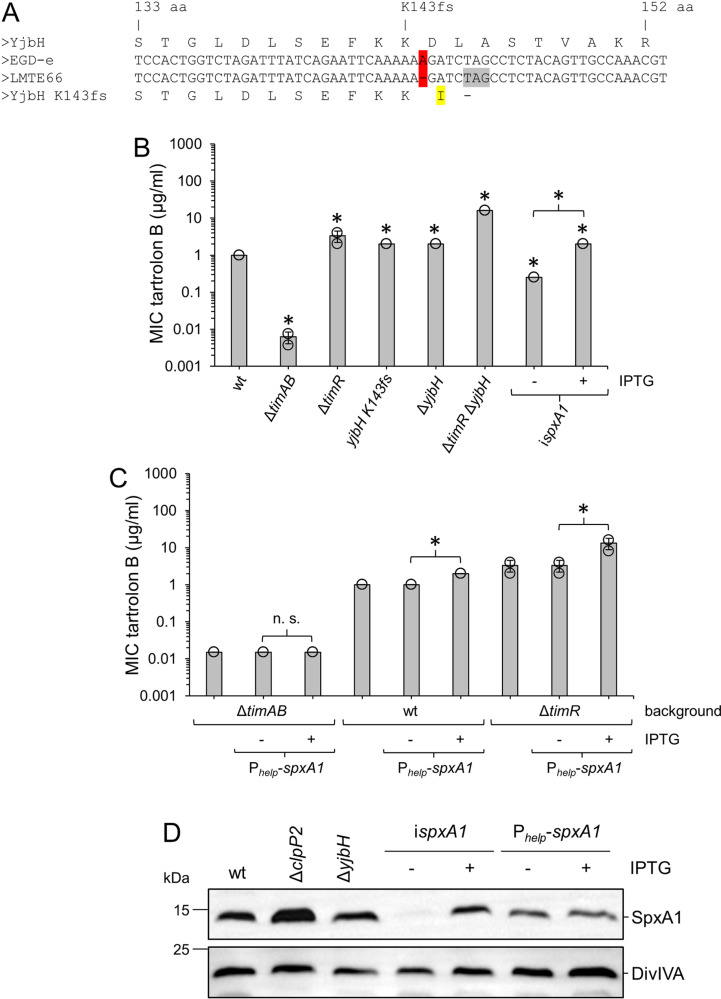
YjbH and SpxA1 contribute to tartrolon B resistance. (A) Inactivation of the *yjbH* gene in the tartrolon B resistant suppressor mutant LMTE66. The deleted nucleotide (red), the frameshifted protein sequence (yellow) and the emerging premature stop codon (grey) are highlighted. (B) Tartrolon B resistance of *yjbH* and *spxA1* mutant strains. Tartrolon B MICs for *L. monocytogenes* strains EDG-e (wt), LMTE34 (Δ*timAB*), LMTE37 (Δ*timR*), LMTE66 (*yjbH K143fs*), LMTE120 (Δ*yjbH*), LMTE141 (Δ*timR* Δ*yjbH*) and LMTE139 (i*spxA1*, ± 1 mM IPTG). (C) Effect of *spxA1* overexpression on tartrolon B resistance in wild type, Δ*timAB* and Δ*timR* backgrounds. *L. monocytogenes* strains used were EGD-e (wt), LMTE34 (Δ*timAB*), LMTE37 (Δ*timR*), LMTE116 (Δ*timAB* P_*help*_*-spxA1*), LMTE117 (P_*help*_*-spxA1*) and LMTE118 (Δ*timR* P_*help*_*-spxA1*). MICs were determined in three independent experiments and average values and standard deviations were calculated. Asterisks mark statistically significant differences compared to wild type (*P*<0.05, *t*-*t*est with Bonferroni-Holm correction for panel B and *P*<0.05, *t*-tes*t* for panel C). (D) SpxA1 levels in *clpP2*, *yjbH* and *spxA1* mutants. Western blots showing SpxA1 (upper blot) and DivIVA levels (for control, bottom blot) in *L. monocytogenes* strains EGD-e (wt), LMTE80 (Δ*clpP2*), LMTE120 (Δ*yjbH*), LMTE139 (i*spxA1*) and LMTE117 (P_*help*_-*spxA1*) grown to mid-logarithmic growth phase in BHI broth ± 1 mM IPTG at 37°C.

Another tartrolon B resistant suppressor (LMTE66) was isolated from EGD-e and this suppressor carried a frameshift mutation in the middle of the *yjbH* gene (*lmo0964*). YjbH is a protease adaptor targeting the redox-responsive transcription factor SpxA1 to ClpXP2 for degradation [20,35]. The 429^th^ bp of *yjbH* was deleted in LMTE66 leading to a frameshift at the 143^th^ amino acid codon and a premature stop codon two codons downstream (Fig 7A). The MIC of tartrolon B for this mutant was two-fold increased (2±0 µg/ml) in compared to wild type (1±0 µg/ml) and a mutant lacking *yjbH* behaved identical (2±0 µg/ml, Fig 7B). Resistance level of a Δ*timR* mutant was approximately three-fold increased in this experiment (3.3±1.2 µg/ml) and therefore was somewhat distinct from the Δ*yjbH* phenotype. However, a 16-fold increase in tartrolon B resistance was observed in a Δ*timR* Δ*yjbH* double mutant (16±0 µg/ml Fig 7B). This suggests that YjbH does not act through TimR and reveals a synergistic relationship of TimR and YjbH instead.

Inspired by these results, we constructed an IPTG-dependent *spxA1* mutant (i*spxA1*, strain LMTE139) that lacks the chromosomal *spxA1* gene but carries an ectopic copy of *spxA1* under control of the IPTG-dependent P_*help*_ promoter [36]. In agreement with previous reports, this mutant required IPTG for growth (S6 Fig) as *spxA1* is an essential gene under aerobic conditions [18,27,37]. Depletion of SpxA1 from strain LMTE139 lowered the MIC of tartrolon B approximately four-fold (0.25±0 µg/ml, Fig 7B), suggesting that tartrolon B resistance and SpxA1 levels directly correlate with each other. To confirm this idea further, the effect of SpxA1 overexpression was determined. SpxA1 overexpression in wild type (using P_*help*_-*spxA1* expressed from an ectopic site) increased tartrolon B resistance two-fold and a fourfold increase was observed upon SpxA1 overexpression in the Δ*timR* background. This latter observation suggests that the synergism of Δ*timR* and Δ*yjbH* deletions discussed above can indeed be explained by increased SpxA1 levels. In contrast, SpxA1 overexpression had no effect in the absence of TimAB (Fig 7C). Thus, tartrolon B cannot be detoxified through accumulation of SpxA1, as long as the compound cannot be exported.

To further test the idea that SpxA1 accumulation plays a role in tartrolon B hyper-resistance of the Δ*clpP2* mutant, we determined SpxA1 levels by Western blotting (Fig 7) followed by densitometry. This demonstrated a 1.8±0.4-fold accumulation of SpxA1 in the Δ*clpP2* mutant compared to the wild type (*P*=0.023, *t*-test, n=3), while the changes in SpxA1 levels in the Δ*yjbH* mutant and during SpxA1 overexpression were insignificant (Figs 7D and S7).

### Possible roles of SpxA1 in tartrolon B resistance

To explain the role of SpxA1 in tartrolon B resistance, we next considered the possibility that SpxA1 could activate *timAB* expression. To test this, we measured activity of the P_*timABR*_ promoter in wild type and a Δ*yjbH* mutant in the absence and the presence of tartrolon B. However, this did not reveal an effect on promoter activity in the Δ*yjbH* deletion strain (S8 Fig). To look at the activity of the fully de-repressed P_*timABR*_ promoter, we also measured P_*timABR*_ promoter activity in a Δ*yjbH* Δ*timR* double mutant, but found it to be similar to a Δ*timR* single mutant (S8 Fig). Thus, YjbH and SpxA1 do not influence activity of the promoter driving *timABR* expression.

Alternatively, tartrolon B treatment could exert an oxidative stress and SpxA1-dependent induction of oxidative stress genes could mitigate this. We investigated this idea by looking for a possible synergism of tartrolon B with hydrogen peroxide in a checkerboard assay. However, this showed that both compounds did not act in a synergistic way (S9 Fig) and, consequently, this second hypothesis was also rejected.

Further to these possibilities, we assumed that SpxA1 could activate genes encoding potassium importers to compensate for tartrolon-induced potassium leakage. If so, tartrolon B resistance should lose its potassium-dependency upon SpxA1 depletion. However, tartrolon B resistance of SpxA1-depleted cells fully responded to the presence of potassium (S1 Table). Thus, this third hypothesis was also rejected. We concluded that there must be a SpxA1-dependent factor, which is unrelated to oxidative stress response, potassium homeostasis and *timABR* transcription, that determines tartrolon B resistance of *L. monocytogenes*.

### Transposon insertion sequencing identifies SpxA1-dependent tartrolon B resistance genes

**Fig 8:**
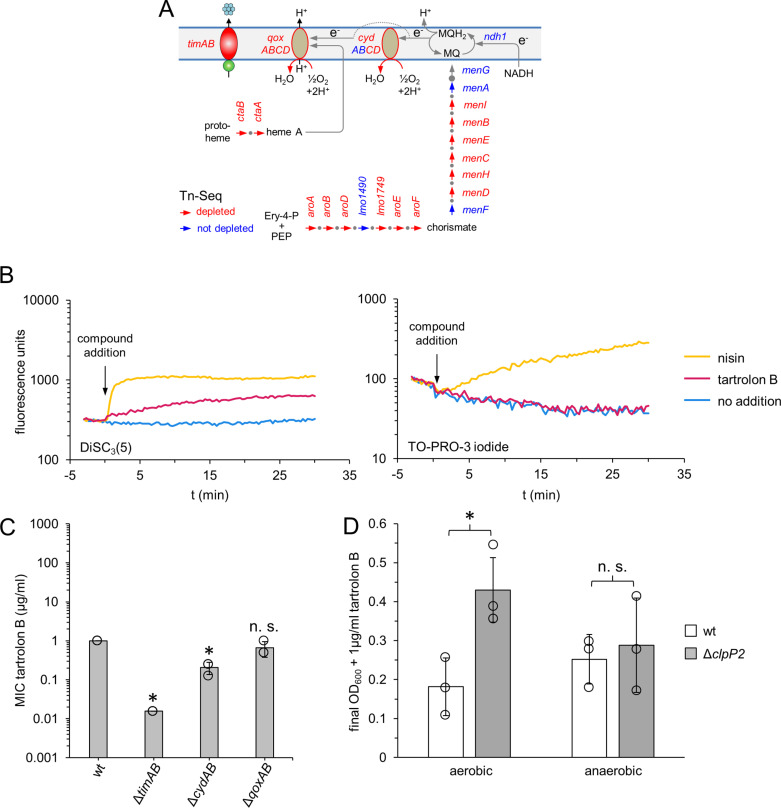
Role of respiratory chain genes for tartrolon B resistance in *Listeria monocytogenes.* (A) Graphic sketch summarising the most important results of the Tn-Seq experiment. Transposon insertions in the *timAB* genes and genes of the shikimate pathway (*aro*), menaquinone (*men*) and heme biosynthesis (*cta*) as well as genes for the assembly of cytochrome oxidases (*cyd*, *qox*) are depleted from a *L. monocytogenes* transposon mutant library after tartrolon B exposure according to Tn-Seq. All but the *timAB* genes are needed for cytochrome oxidase activity. Abbreviations: Ery-4-P – erythose-4-phosphate; PEP – phosphoenolpyruvate; MQ – menaquinones; NADH – nicotinamide adenine dinucleotide; e⁻ – electron. (B) Tartrolon B dissipates the membrane potential without pore formation. Cells of *L. monocytogenes* wildtype strain EGD-e were stained with the membrane potential sensitive dye DiSC_3_(5) (left panel) and the membrane-impermeable DNA stain TO-PRO-3 iodide (right panel). The stained cells were then challenged with tartrolon B at 1 x MIC (1 µg/ml) or for control with nisin at 1 x MIC (1 mg/ml) and changes in fluorescence emission were recorded. (C) Effect of a *cydAB* and *qoxAB* deletions on tartrolon B resistance of *L. monocytogenes*. *L. monocytogenes* strains used were EGD-e (wt), LMTE34 (Δ*timAB*), LMSW246 (Δ*cydAB*) and LMSW235 (Δ*qoxAB*). Average values and standard deviations were calculated from three repetitions. Asterisks mark statistically significant differences compared to wild type (*P*<0.01, *t*-*t*est with Bonferroni-Holm correction, n. s. – not significant). (D) The tartrolon B resistant phenotype of the Δ*clpP2* mutant is oxygen-dependent. Final optical densities of cultures of *L. monocytogenes* strains EGD-e (wt) and LMTE80 (Δ*clpP2*) cultivated in BHI broth + 1 µg/ml tartrolon B (= 1 x MIC for the wild type) and grown in microplates at 37°C for 24 hours either in the presence (aerobic) or absence of oxygen (anaerobic). The experiment was repeated three times and average values and standard deviations are shown. Statistical significance is indicated (* – *P*<0.05, *t*-*t*est, n. s. – not significant).

To identify the SpxA1-dependent factor(s) that contribute to tartrolon B resistance, we searched for genes that sensitize *L. monocytogenes* against tartrolon B when mutated. For this, we challenged a transposon insertion library, previously generated to facilitate transposon insertion sequencing (Tn-Seq) in strain EGD-e [27], with a sub-inhibitory concentration of tartrolon B (0.25 µg/ml). Transposon insertion mutants in *timA* and *timB* were the most strongly depleted mutants during tartrolon B exposure (Table 1), validating the Tn-Seq approach. However, a specific pattern of tartrolon B-sensitive mutants was discovered at the same time (Tables 1 and S2). This pattern consisted of most shikimate pathway genes, the majority of the menaquinone biosynthesis genes, genes encoding the subunits of the two cytochrome oxidases as well as several heme biosynthesis genes. Additionally, transposon insertion mutants in genes needed for lipoteichoic acid biosynthesis, in genes required for activation of the alternative sigma factor σ^B^ and in genes belonging to several other functional categories were depleted (Table 1). The shikimate pathway generates chorismate to fuel menaquinone biosynthesis. Menaquinone is required to transfer electrons from NADH to cytochrome oxidases that reduce molecular O_2_ to water and contribute to the generation of a transmembrane proton gradient (Fig 8A) and these cytochrome oxidases are dependent on heme as prosthetic groups [38]. Depletion of transposon mutants in these groups of genes indicates that the functionality of the respiratory chain is of special importance for resistance to tartrolon B. Moreover, of the 58 genes identified by Tn-Seq, only four were known SpxA1-dependent genes (Table 1), and out of these, the *cydCD* genes coding for an ABC transporter required for the function of the cytochrome oxidase CydAB [39], which is encoded in the same operon [40], showed the strongest SpxA1-dependence in the work of Cesinger *et al.* [41]. Cytochrome oxidases are required for the maintenance of proton motive force (PMF) [42] and thus for ATP biosynthesis by F0/F1-type ATPases. Interestingly, treatment with tartrolon B led to a release of the voltage-dependent dye DiSC_3_(5) from the membrane as a sign of membrane depolarization. However, tartrolon B did not affect membrane integrity, which is typical for pore-forming compounds such as nisin (Fig 8B). Membrane depolarization by tartrolon B without pore formation suggests that the compound also could affect the transmembrane proton gradient, presumably as a protonophore. Therefore, we reasoned that SpxA1-mediated activation of *cydABCD* expression could be beneficial to strengthen PMF during tartrolon B exposure and generated a Δ*cydAB* mutant to determine the role of the CydAB cytochrome oxidase in tartrolon B resistance. As can be seen in Fig 8C, tartrolon B resistance of the Δ*cydAB* mutant was indeed reduced fivefold. In contrast, *qoxAB* deletion was without significant effect on tartrolon B resistance in this MIC assay, which is in good agreement with the milder depletion of *qox* mutants in the Tn-Seq experiment (Tables 1 and S2). These results support the idea that the tartrolon B hyper-resistance of the Δ*clpP2* mutant is related to SpxA1-dependent activation of the *cydABCD* operon. To test this, we tried to combine *cydAB* or *spxA1* mutations with the Δ*clpP2* deletion, but were unsuccessful despite repeated attempts. As an alternative, we asked whether the tartrolon B-resistant phenotype of the Δ*clpP2* mutant is dependent on the presence of oxygen, which would logically follow from the dependence on cytochrome oxidases. In fact, the Δ*clpP2* mutant grew to higher final optical densities in the presence of 1 µg/ml tartolon B than the wild type under aerobic conditions, reflecting its resistance, but no such effect was observed in the absence of oxygen (Fig 8D).

**Table 1:** Genes required for tartrolon B resistance according to Tn-Seq.

locus	name	function	fold change^1^	p^2^	SpxA1 control^3^
**Tartrolon B export**					
*lmo1964*	*timA*	tartrolon B ABC exporter, ATP-binding protein	25.89	3.2x10^-6^	no
*lmo1963*	*timB*	tartrolon B ABC exporter, membrane protein	21.42	2.6x10^-7^	no
**Shikimate pathway**					
*lmo1749*	*–*	shikimate kinase	5.38	5.4x10^-3^	no
*lmo0491*	*aroD*	3-dehydroquinate dehydratase	4.71	1.2x10^-3^	no
*lmo1600*	*aroA*	3-deoxy-D-arabino-heptulosonate 7-phosphate synthase	3.36	4.6x10^-2^	no
*lmo1927*	*aroB*	3-dehydroquinate synthase	3.21	4.5x10^-3^	no
*lmo1928*	*aroF*	chorismate synthase	3.19	5.2x10^-3^	no
*lmo1923*	*aroE*	3-phosphoshikimate 1-carboxyvinyltransferase	2.86	5.2x10^-3^	no
**Menaquinone biosynthesis**					
*lmo2385*	*menI*	DHNA-CoA Thioesterase	11.00	1.1x10^-2^	no
*lmo1672*	*menE*	O-succinylbenzoic acid--CoA ligase	3.53	1.2x10^-2^	no
*lmo1931*	*menH*	ubiquinone/menaquinone biosynthesis methyltransferase	3.51	7.1x10^-3^	no
*lmo1675*	*menD*	2-succinyl-5-enolpyruvyl-6-hydroxy-3-cyclohexene-1-carboxylate synthase	3.24	5.1x10^-4^	no
*lmo1673*	*menB*	naphthoate synthase	3.06	3.5x10^-2^	no
*lmo2520*	*menC*	O-succinylbenzoate-CoA synthase	2.93	4.3x10^-3^	no
**Heme biosynthesis**					
*lmo1553*	*hemL*	glutamate-1-semialdehyde aminotransferase	9.70	5.8x10^-4^	no
*lmo2057*	*ctaB*	heme O synthase	2.49	8.5x10^-3^	no
*lmo2058*	*ctaA*	heme A synthase	2.45	4.6x10^-3^	no
**Cytochrome oxidases**					
*lmo2715*	*cydD*	ABC transporter required for cytochrome *bd* assembly	6.19	3.3x10^-2^	yes
*lmo2716*	*cydC*	ABC transporter required for cytochrome *bd* assembly	4.95	6.8x10^-4^	yes
*lmo0015*	*qoxC*	AA3-600 quinol oxidase subunit III	2.64	1.1x10^-3^	no
*lmo0016*	*qoxD*	AA3-600 quinol oxidase subunit IV	2.54	1.2x10^-3^	no
*lmo0013*	*qoxA*	AA3-600 quinol oxidase subunit II	2.43	2.7x10^-3^	no
*lmo0656*	*ctaM*	required for biogenesis of cytochrome oxidase	2.23	8.0x10^-3^	yes
*lmo0014*	*qoxB*	AA3-600 quinol oxidase subunit I	2.20	2.6x10^-3^	no
**Control of SigB activity**					
*lmo0894*	*rsbW*	negative regulator RsbW	6.23	3.4x10^-2^	no
*lmo0895*	*sigB*	alternative sigma factor	6.22	1.8x10^-5^	no
*lmo0891*	*rsbT*	protein serine kinase	5.32	2.9x10^-4^	no
*lmo0890*	*rsbS*	part of the stressosome	4.66	6.3x10^-5^	no
*lmo0892*	*rsbU*	protein serine phosphatase	4.57	3.2x10^-4^	no
*lmo0893*	*rsbV*	anti-anti-sigma factor (antagonist of RsbW)	4.20	1.9x10^-4^	no
*lmo0889*	*rsbR*	stressosome sensor protein	3.79	1.9x10^-3^	no
**Regulation of gene expression**					
*lmo2515*	*degU*	orphan response regulator	3.89	2.7x10^-2^	no
*lmo0051*	*agrA*	quorum sensing response regulator	2.42	1.3x10^-3^	no
*lmo0050*	*agrC*	quorum sensing histidine kinase	2.38	9.0x10^-4^	no
*lmo0651*	*mouR*	transcriptional regulator, GntR family	2.37	6.6x10^-4^	no
**Protein biosynthesis**					
*lmo2653*	*tuf*	elongation factor Tu	11.00	2.1x10^-3^	no
*lmo1596*	*rpsD*	ribosomal protein S4	5.00	4.7x10^-2^	no
*lmo1843*	*ylyB*	putative pseudouridylate synthase	4.23	1.6x10^-2^	no
*lmo0256*	*–*	ribosomal RNA small subunit methyltransferase C	3.16	6.2x10^-4^	no
*lmo1366*	*yqxC*	similar to rRNA methyltransferase	2.70	1.3x10^-2^	no
*lmo1325*	*infB*	translation initiation factor IF-2	2.37	1.7x10^-3^	no
*lmo0866*	*cshA*	DEAD-box RNA helicase	2.33	1.6x10^-2^	no
*lmo1473*	*dnaK*	class I heat-shock protein (molecular chaperone) DnaK	2.00	2.6x10^-2^	no
**Lipoteichoic acid biosynthesis**					
*lmo0644*	*ltaP*	LTA primase	5.88	7.5x10^-5^	yes
*lmo2477*	*galE*	UDP-glucose 4-epimerase	3.47	1.3x10^-3^	no
*lmo2554*	*lafB*	LTA anchor formation protein B	3.01	4.8x10^-3^	no
*lmo2555*	*lafA*	LTA anchor formation protein A	2.25	8.9x10^-3^	no
**Other functional categories**					
*lmo2769*	*eslA*	ABC transporter, ATP-binding protein	4.84	1.2x10^-3^	no
*lmo2768*	*eslB*	ABC transporter, permease component	4.56	1.4x10^-3^	no
*lmo0052*	*pdeA*	c-di-AMP specific phosphodiesterase	2.03	1.3x10^-2^	no
*lmo1293*	*glpD*	glycerol-3-phosphate dehydrogenase	2.24	3.2x10^-3^	no
*lmo2072*	*recR*	required for the formation of RecA DNA repair centers	3.83	1.1x10^-3^	no
*lmo0006*	*gyrB*	DNA gyrase subunit B	3.00	1.6x10^-2^	no
*lmo0289*	*–*	YycH protein	4.75	1.7x10^-3^	no
*lmo2231*	*–*	putative cobalt-zinc-cadmium resistance protein	2.57	7.8x10^-3^	no
*lmo1495*	*–*	conserved hypothetical protein	2.35	3.5x10^-2^	no
*lmo1974*	*–*	putative transcriptional regulator, GntR family	2.05	1.5x10^-2^	no
*lmo2229*	*pbpA2*	penicillin binding protein A2	2.61	2.3x10^-2^	no

^1^$\text{fold change}=\frac{\sum_{n=1}^{3}\left(numberofTn-Seqreadspergeneinuntreatedcontrol\right)}{\sum_{n=1}^{3}\left(numberofTn-SeqreadspergeneintartrolonBtreatedculture\right)}$, numbers of Tn-Seq reads per gene for each replicate and condition can be found in S2 Table.

^2^*p*-value according to *t*-test (n=3).

^3^SpxA1-dependent transcriptional activation according to published work [41].

## Discussion

### The membrane is the site of attack and defence against tartrolon B

Tartrolon B is a potassium ionophore [5,13]. Repeated cycles of potassium binding to tartrolon B on the cytosolic side and their release on the extracellular side of the cytoplasmic membrane breaks down the concentration gradient between high internal and low external potassium concentrations. Detoxification of tartrolon B by the TimAB MDR transporter is a recently discovered mechanism of tartrolon B resistance [5]. How TimAB detoxifies tartrolon B has not been investigated, but TimAB likely removes lipohilic tartrolon B from the membrane in analogy to the hydrophobic vacuum cleaner concept developed for other ABC-type MDR transporters such as *Lactococcus lactis* LmrA [43]. These transporters are able to detoxify drugs that intercalate in the membrane and extrude them directly from the membrane compartment to release them to the extracellular space without intermediate transport steps through the cytoplasm [44]. Thus, the main layer of defense is organized by TimAB at the cytoplasmic membrane, even though trace amounts of the compound must also reach the cytoplasm, where TimR senses tartrolon B and only would stop to repress *timABR* expression upon direct compound contact [5].

### ClpP2-containing proteasomes contribute to tartrolon B resistance

The data that we present here reveal a higher complexity in the steps needed to establish full tartrolon B resistance. Suppressor screens and follow-up genetic experiments demonstrated that the ClpCP2 and ClpXP2 proteasomes are involved in the detoxification of tartrolon B. Both proteasomes share critical functions in virulence, since deletion of either *clpC*, *clpX* or *clpP2* impaired replication of *L. monocytogenes* in the liver and spleens of infected mice [28,32,34,45]. However, both proteasomes also have specific functions: ClpC controls peptidoglycan (PG) biosynthesis through regulated degradation of MurA, the first committed step enzyme of this pathway [24,26,46,47], but no such role has been disclosed so far for ClpX. Instead, ClpX has been ascribed a role in control of cellular SpxA1 amounts by mediating its proteolytic degradation [34]. SpxA1, a transcription factor, interacts with YjbH [35], an adaptor for ClpX containing proteasomes in *L. monocytogenes* and *B. subtilis* [20,35,48], and is degraded by ClpXP in the presence of YjbH [20,48]. However, even this function is shared between ClpC and ClpX, as *B. subtilis* Spx is also degraded by ClpCP *in vitro* as long as suitable adapters (MecA or YpbH) are present [19]. Our results would also be congruent with the idea that ClpCP2 and ClpXP2 proteasomes both contribute to SpxA1 degradation *in vivo*, as tartrolon B hyper-resistance observed in a mutant lacking *clpP2* can be phenocopied by simultaneous deletion of *clpC* and *clpX*. When these two proteolytic machines are non-functional, either because their shared protease, their associated ATPases or adaptor proteins have been deleted, SpxA1 would accumulate. That SpxA1 overproduction resulted in intermediate tartrolon B resistance levels only, could be explained by ongoing SpxA1 degradation in the strains used for *spxA1* overexpression. Lack of SpxA1 degradation might be the dominating influence on SpxA1 levels compared to artificially increased expression. In good agreement, depletion of SpxA1 impaired tartrolon B resistance and, thus, accumulation of SpxA1 in strains with dysfunctional proteolysis is the most plausible explanation for their tartrolon B hyper-resistance.

### The SpxA1-dependent *cydABCD* operon is a tartrolon B resistance determinant

**Fig 9:**
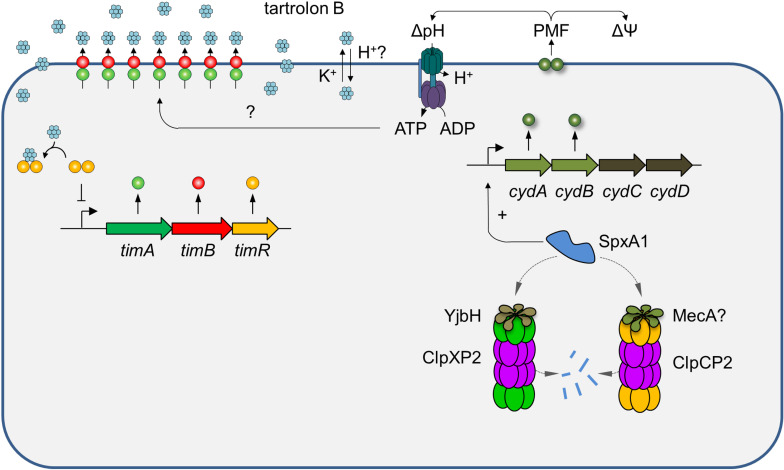
Mechanisms maintaining and regulating tartrolon B resistance in *L. monocytogenes.* The TimAB transporter exports tartrolon B, but *timABR* transcription is repressed by TimR as long as tartrolon B is absent. Tartrolon B is a specific inducer of the *timABR* operon as it binds to TimR leading to loss of *timABR* repression [5]. ClpCP2 and ClpXP2 proteasomes support tartrolon B resistance by controlling proteolytic stability of SpxA1. Proteolytic degradation of SpxA1 by ClpXP2 depends on the YjbH adaptor protein while ClpCP2 presumably requires MecA for SpxA1 degradation. SpxA1 activates expression of the *cydABCD* operon [41], which encodes the genes for the CydAB cytochrome oxidase and CydCD, which is required for CydAB maturation. CydAB generates a proton gradient that can either energize TimAB or may help to counteract dissipation of the transmembrane proton gradient by tartrolon B, which potentially acts as a coupled potassium ionophore/protonophore. PMF – proton motiv force.

Remarkably, the effects observed on tartrolon B resistance upon *clpP2* deletion and SpxA1 overproduction were both TimAB-dependent, but obvious models explaining this TimAB-dependence could be ruled out, as neither TimA stability nor P_*timABR*_ promoter activity depended on ClpP2. We therefore hypothesized that a SpxA1-controlled gene must support tartrolon B resistance. Genome wide identification of genes that determine the resistance to tartrolon B using Tn-Seq identified specific signature genes. Among these were most genes of the shikimate and menaquinone biosynthesis pathway, several heme biosynthesis genes (*hemL*, *ctaA, ctaB*), two genes (*cydCD*) required for assembly of the CydAB *bd*-type cytochrome [49], and the four genes for the subunits of the cytochrome *aa*_*3*_-type menaquinol oxidase (*qoxABCD*). The shikimate pathway generates chorismate as a menaquinone precursor and heme biosynthesis provides the prosthetic groups of cytochrome oxidases. Thus, all these pathways are essential to maintain the function of the respiratory chain, in particular the function of the two cytochrome oxidases CydAB and QoxABCD. Transcription of the *cydABCD* operon is strongly SpxA1-dependent [41]. That only the *cydCD* genes but not the *cydAB* genes were found in the Tn-Seq screens can be explained by the strong growth defect of a Δ*cydAB* mutant [38]. We generated a Δ*cydAB* mutant and found that this mutant was indeed more susceptible against tartrolon B. Cytochrome oxidases transfer electrons from menaquinol to molecular oxygen and either pump protons out of the cell (QoxABCD) or contribute to transmembrane charge separation (CydAB), which both promote the generation of proton motive force (PMF), an electrochemical gradient made up of the proton concentration gradient (ΔpH) and the resulting electrical potential difference (ΔΨ, Fig 9) [38,50,51]. Cytochrome oxidase activity could be crucial for PMF maintenance when ionophores such as tartrolon B selectively increase the permeability of the membrane. Given the specific function of cytochrome oxidases in PMF generation and the observed depolarization of *L. monocytogenes* membranes by tartrolon B, it seems plausible that tartrolon B does not only transport potassium ions out of the cell, but in return also could transport protons into the cell. Tartrolon B could thus act as a coupled protonophore/ionophore that dissipates potassium and proton gradients across the cytoplasmic membrane, a mode of action, also observed with other antimicrobial compounds such as bedaquiline [52] and nigericin [53]. In such a scenario, counteracting the dissipation of the proton gradient through transcriptional activation of cytochrome oxidase genes would explain the tartrolon B hyper-resistance of the Δ*clpP2* mutant. The fourfold increased nigericin resistance of the Δ*clpP2* mutant (S2 Fig) would support this idea. Because the cell only responds to dissipation of the proton gradient by tartrolon B using this strategy, TimAB would still be required for tartrolon B detoxification and thus this effect remains *timAB*-dependent. Alternatively, activation of *cydABCD* expression in mutants with higher SpxA1 levels could promote the generation of additional ATP, needed for energizing TimAB, through proton gradient driven F_0_/F_1_-type ATPases (Fig 9). This hypothesis would also be in good agreement with the observed TimAB-dependence of the Δ*clpP2* mutant´s tartrolon B hyper-resistance. However, we do not observe hyper-resistance of the Δ*clpP2* mutant against antimicrobial compounds exported by other ABC-type drug exporters such as aurantimycin A (S2 Fig). This challenges the idea that the activation of cytochrome oxidases is a general strategy to supply ABC-transporters with additional energy. Lastly, tartrolon B could directly inhibit cytochrome oxidases, as shown for several other antimicrobial compounds [49] and overexpression of *cydABCD* in the Δ*clpP2* mutant would counteracts this effect. However, we think that this is less likely since the Δ*cydAB* mutant is sensitive and not resistant to tartrolon B.

Notwithstanding these open questions, our work has disclosed a so far unknown role for the Clp/SpxA1 pathway and the *cydABCD* operon as one of its targets in the resistance against tartrolon B. Furthermore, it indicates a dual mode of action for tartrolon B. We believe that studying the mechanisms that drive resistance to such understudied antimicrobial compounds has the potential to provide new insights into the mechanisms by which bacterial cells maintain their growth and integrity under conditions that mimic the selective pressures in their natural habitats.

## Materials and methods

### Bacterial strains and growth conditions

All strains and plasmids used in this study are listed in Table 2. *L. monocytogenes* strains were grown in BHI broth or on BHI agar plates at 37°C. Erythromycin (5 µg ml^-1^), kanamycin (50 µg ml^-1^), X-Gal (50 µg ml^-1^) or IPTG (1 mM) were added when required. *Escherichia coli* TOP10 was used as the standard cloning host [54]. For growth under anaerobic conditions, cultures were grown in a 0.5% O_2_ atmosphere using a Whitley H35 hypoxystation. Tartrolon B was obtained from the DZIF natural compound selection, boromycin was purchased from Hello Bio Ltd (Ireland), ADEP2 was procured from biomol GmbH (Germany) and nigericin was obtained from Sigma-Aldrich (Germany).

**Table 2:** Plasmids and strains used in this study.

name	relevant characteristics	source/ reference
**plasmids**		
pET11a	*bla* P_*T7*_ *lacI*	Novagen
pHoss1	*bla erm tetR* P_*xyl/tetO*_*-anti-secY*	[55]
pIMK3	P_*help*_*-lacO lacI neo*	[36]
pMAD	*bla erm bgaB*	[56]
pminiMAD	*bla erm*	[57]
pJR127	*bla erm bgaB* Δ*clpC (lmo0232)*	[26]
pTE16	P_*timABR*_*-lacZ neo*	[5]
pTE61	P_*help*_*-lacO-timAB lacI neo*	[5]
pPR2	*bla erm bgaB* Δ*clpP1* (*lmo1138*)	this work
pPR5	P_*help*_*-lacO-clpP2 lacI neo*	this work
pPR8	*bla erm bgaB* Δ*clpP2* (*lmo2468*)	this work
pSW54	*bla* P_*T7*_ *lacI clpP2-strep*	this work
pSW128	*bla erm bgaB* ∆*qoxAB (lmo0013-lmo0014)*	this work
pSW129	*bla erm tetR* P_*xyl/tetO*_*-anti-secY* ∆*cydAB* (*lmo2718-lmo2717*)	this work
pTE64	*bla* P_*T7*_ *lacI clpP-strep E9K*	this work
pTE87	*bla erm* Δ*clpP2 (lmo2468)*	this work
pTE97	*bla erm bgaB* Δ*clpX (lmo1268)*	this work
pTE99	*bla erm bgaB* Δ*clpE (lmo0997)*	this work
pTE103	*bla erm* Δ*clpE*	this work
pTE107	P_*help*_*-lacO-timA-strep-timB lacI neo*	this work
pTE111	*bla erm tetR* P_*xyl/tetO*_*-anti-secY* Δ*clpX*	this work
pTE115	P_*help*_*-lacO-spxA1 lacI neo*	this work
pTE116	*bla erm bgaB* Δ*yjbH* (*lmo0964*)	this work
pTE117	*bla erm bgaB* Δ*spxA1* (*lmo2191*)	this work
pTE118	*bla erm* Δ*spxA1*	this work
*L* ***. monocytogenes* strains**		
EGD-e	wild type	[58]
LMS250	Δ*hly*	[27]
LMJR138	Δ*clpC*	[26]
LMSH16	*attB::lacZ neo*	[10]
LMSH98	Δ*lftR attB::*P_*lieAB*_*-lacZ neo*	[14]
LMTE19	*attB::*P_*timABR*_*-lacZ neo*	[5]
LMTE34	Δ*timAB*	[5]
LMTE37	Δ*timR*	[5]
LMTE50	Δ*timR attB::*P_*timABR*_*-lacZ neo*	[5]
LMPR6	Δ*clpP1*	pPR2 ↔ EGD-e
LMPR7	*attB::*P_*help*_*-lacO-clpP2 lacI neo*	pPR5 → EGD-e
LMSW235	∆*qoxAB*	pSW128 ↔ EGD-e
LMSW246	∆*cydAB*	pSW129 ↔ EGD-e
LMTE38	*attB::*P_*timABR*_*-lacZ neo clpP2 E9K*	tartB^R^ suppressor of LMTE19
LMTE39	*attB::*P_*timABR*_*-lacZ neo clpP2 E9K*	tartB^R^ suppressor of LMTE19
LMTE40	*attB::*P_*timABR*_*-lacZ neo clpP2 E9K*	tartB^R^ suppressor of LMTE19
LMTE42	*attB::*P_*timABR*_*-lacZ neo clpP2 E9K*	tartB^R^ suppressor of LMTE19
LMTE43	*attB::*P_*timABR*_*-lacZ neo clpP2 E9K*	tartB^R^ suppressor of LMTE19
LMTE44	*attB::*P_*timABR*_*-lacZ neo clpP2 E9K*	tartB^R^ suppressor of LMTE19
LMTE45	*attB::*P_*timABR*_*-lacZ neo clpP2 E9K*	tartB^R^ suppressor of LMTE19
LMTE46	*attB::*P_*timABR*_*-lacZ neo clpP2 E9K*	tartB^R^ suppressor of LMTE19
LMTE47	*attB::*P_*timABR*_*-lacZ neo clpP2 E9K*	tartB^R^ suppressor of LMTE19
LMTE48	*attB::*P_*timABR*_*-lacZ neo clpP2 E9K*	tartB^R^ suppressor of LMTE19
LMTE49	*attB::*P_*timABR*_*-lacZ neo clpP2 E9K*	tartB^R^ suppressor of LMTE19
LMTE65	*timR K202fs*	tartB^R^ suppressor of EGD-e
LMTE66	*yjbH K143fs*	tartB^R^ suppressor of EGD-e
LMTE74	*clpP2 T90I lmo1296 G241S*	tartB^R^ suppressor of EGD-e
LMTE75	*clpP2 T90I lmo1296 G241S*	tartB^R^ suppressor of EGD-e
LMTE76	*clpP2 T90I lmo1296 G241S*	tartB^R^ suppressor of EGD-e
LMTE80	Δ*clpP2*	pTE87 ↔ EGD-e
LMTE89	*attB::*P_*help*_*-lacO-timAB lacI neo*	pTE61 → EGD-e
LMTE90	Δ*clpP2 attB::*P_*help*_*-lacO-clpP2 lacI neo*	pTE87 ↔ LMPR7
LMTE91	P_*timABR*_*G-14T attB::*P_*help*_*-lacO-clpP2 lacI neo*	tartB^R^ suppressor of LMPR7
LMTE94	Δ*timR attB::*P_*help*_*-lacO-timAB lacI neo*	pTE61 → LMTE37
LMTE95	Δ*clpP2* Δ*timAB*	pTE87 ↔ LMTE34
LMTE100	Δ*clpE*	pTE103 ↔ EGD-e
LMTE102	Δ*clpE* Δ*clpC*	pJR127 ↔ LMTE100
LMTE106	Δ*timAB attB::*P_*help*_*-lacO-timA-strep-timB lacI neo*	pTE107 → LMTE34
LMTE110	Δ*clpP2* Δ*timAB attB::*P_*help*_*-lacO-timA-strep-timB lacI neo*	pTE87 ↔ LMTE106
LMTE112	Δ*clpE* Δ*clpX*	pTE111 ↔ LMTE100
LMTE113	Δ*clpX*	pTE111 ↔ EGD-e
LMTE114	Δ*clpC* Δ*clpX*	pJR127 ↔ LMTE113
LMTE115	Δ*clpC* Δ*clpE* Δ*clpX*	pTE111 ↔ LMTE102
LMTE116	Δ*timAB attB::*P_*help*_*-lacO-spxA1 lacI neo*	pTE115 → LMTE34
LMTE117	*attB::*P_*help*_*-lacO-spxA1 lacI neo*	pTE115 → EGD-e
LMTE118	Δ*timR attB::*P_*help*_*-lacO-spxA1 lacI neo*	pTE115 → LMTE37
LMTE120	Δ*yjbH*	pTE116 ↔ EGD-e
LMTE139	Δ*spxA1 attB::*P_*help*_*-lacO-spxA1 lacI neo*	pTE118 ↔ LMTE117
LMTE141	Δ*timR* Δ*yjbH*	pTE116 ↔ LMTE37
LMTE142	Δ*timR* Δ*yjbH attB::*P_*timABR*_*-lacZ neo*	pTE16 → LMTE141
LMTE147	Δ*yjbH attB::*P_*timABR*_*-lacZ neo*	pTE16 → LMTE120

*The arrow (→) stands for a transformation event and the double arrow (↔) indicates gene deletions obtained by chromosomal insertion and subsequent excision of pMAD/pMiniMAD/pHoss1 plasmid derivatives (see experimental procedures for details).

### General methods, manipulation of DNA and oligonucleotide primers

Standard methods were used for transformation of *E. coli*, for isolation of plasmid DNA and for PCR [54]. Transformation of *L. monocytogenes* was carried out as described by others [36]. Restriction and ligation of DNA was performed following the manufacturer´s instructions. All primer sequences are listed in Table 3.

**Table 3:** Primers used in this study.

name	sequence (5´→3´)
MF38	GTCTCGTGGGCTCGGAGATGTGTATAAGAGACAGNN
MF52	5´-phosphate-CTGTCTCTTATACACATCTCCGAGCCCACGAGAC-3´-phosphate
MF53	TCGTCGGCAGCGTCAGATGTGTATAAGAGACAGCGAGGAATTTGTATCG
PR1	CTAGACAGATCTATCGATGCATGCCATGGGCAGATTGCTCGTCTAACAGCG
PR2	GCCTCGCGTCGGGCGATATCGGATCCCGGATTATTACCAAAATCGTTCTC
PR3	GATATGGTCGACTAATAAAAAAAGAGGTTTTGCACAAAATG
PR4	TTATTAGTCGACCATATCAAGATTCCTCCTGTTAAATG
PR5	TATCGATGCATGCCATGGAACTAAAAACAATCAACGTATCTCTCGTAT
PR6	TCGGGCGATATCGGATCCGATTCTTGTCCCTTGATGAAAAGTAAGT
PR7	TTCATGGTCGACTAAAACCAAAAGGTTCACTTCTTTTTGT
PR8	GTTTTAGTCGACCATGAAAAAATTCCTCCTTAAAAAGCCTTAGTTT
PR19	CATG GAAAAGGATCCG AACTTAATTCCAACAGTAATTGAA
PR20	AATGGGATCGTCGACTTAGCCTTTTAAGCCAGAT
SHW401	GCGCACTAGTCATATGTATATCTCCTTCTTAAAG
SHW402	CGCGCTCGAGCAAAGCCCGAAAGGAAGCTG
SW134	GCGCGCACTAGTATGAACTTAATTCCAACAGTAATTGAACAAAC
SW135	CGCGCGCTCGAGTTATTTTTCGAACTGCGGGTGGCTCCAGCCTTTTAAGCCAGATTTATTAATGATAATATC
SW264	GAAGCTTCTGCAGACGCGTCGACCGAAACATTAGAAGAACCAGTGCC
SW265	TTTACACCCGAATCCCCTCTTTTCC
SW268	GATTCGGGTGTAAATGGAATCTGTAGAAACAAATAAAAATCTGC
SW269	CGCGTCGGGCGATATCGGATCCCTTCTGTCATATGCATGAACATCAG
TE237	CTTAATTCCAACAGTAATTAAACAAACTAGCCGCGGTG
TE238	CACCGCGGCTAGTTTGTTTAATTACTGTTGGAATTAAG
TE379	TGGAGCCACCCGCAGTTCGAAAAATACGAAGGGGGAGAAATCAAG
TE380	TTTTTCGAACTGCGGGTGGCTCCATAATGTCATAAATTTATCTTCCAGCTC
TE387	GGCGATATCGGATCCATATGACGTCGACTGCACGCACAAATGGAAAAAG
TE388	GATCTATCGATGCATGCCATGGTACCCGGGGGATCGCCTTCTGCATCAGTGG
TE389	GAAATATTTTGCTGCAGTGATTAGAATGCGCGTCGTAATTG
TE390	CATTCTAATCACTGCAGCAAAATATTTCACCCCTTCGC
TE391	GGCGATATCGGATCCATATGACGTCGACCCAAGAAATTGGGAGCTTCTGG
TE392	GATCTATCGATGCATGCCATGGTACCCGGGCTACTTATCGTTGCCATCATC
TE393	CCTTTTTATTACTGCAGCATATATAATTTCCTCCTTTTAAAAATGAG
TE394	AATTATATATGCTGCAGTAATAAAAAGGGCTTGAGTTAAC
TE410	CAAGCTTGCATGCCTGCAGGTCGACCTGGGGAAGCCTAACTGGTGGC
TE411	GCCAGTGAATTCGAGCTCGGTACCGAATCAATCATGAAGACTTACAAG
TE412	GTGCTTCTTTTTTACTGCAGCATTTGCTATCACCTGATTTTC
TE413	GCAAATGCTGCAGTAAAAAAGAAGCACCCATTCCTGG
TE416	CAAGCTTGCATGCCTGCAGGTCGACGACTTGTATCCAGAGCGGACCTTC
TE417	GCCAGTGAATTCGAGCTCGGTACCGTAAAACATATGATCCGACGTCTC
TE418	CAATCTTACTGCAGCATTAGACATTCACACTCCTTATC
TE419	GTGTGAATGTCTAATGCTGCAGTAAGATTGATAAAAGTGGTTGCC
TE452	GGAGAGTGAAACCCATGGTAACGTTATACACTTCACC
TE453	GCTTTGGTCGACTTAGTTAACCATTTTTTGCGCTTC
TE475	CTATCGATGCATGCCATGGTACCCGGGGGAATCCGAAAGCAGTGAAATG
TE480	GAGAAAAGGGTGCTGCAGATGGGAAAAGATTTAAGGAACTAC
TE481	GGCGATATCGGATCCATATGACGTCGACGCCATATTCATCATTTTAGGTAAG
TE482	CTTAAATCTTTTCCCATCTGCAGCACCCTTTTCTCCTCCTTTTTG

### Construction of bacterial plasmids and strains

Plasmid pSW54 was constructed for overexpression of *clpP2* in *Escherichia coli*. For this purpose, the *clpP2* gene was amplified using oligonucleotides SW134/SW135, cut SpeI/XhoI and ligated into the SpeI/XhoI cut backbone of pET11a, which had been linearized before in a PCR using the oligonucleotides SHW401/SHW402. The E9K mutation was later introduced into pSW54 using quikchange PCR and the oligonucleotide pair TE237/TE238 as the primers, resulting in plasmid pTE64.

Plasmid pPR2 was generated for deletion of *clpP1*. Regions up- and downstream of *clpP1* were amplified using the primer pairs PR5/PR8 and PR7/PR6, respectively, fused together by splicing by overlapping extension (SOE) PCR and the resulting fragment was inserted into pMAD using BamHI/NcoI.

For deletion of *clpP2*, we initially constructed plasmid pPR8. To this end, fragments up- and downstream of *clpP2* were amplified with PR1/PR4 and PR3/PR2, respectively, connected with each other by splicing by overlapping extension PCR (SOE-PCR) and inserted in pMAD using restriction free (RF) cloning. As all attempts to use plasmid pPR8 for *clpP2* deletion were unsuccessful for unknown reasons, the Δ*clpP2* allele of pPR8 was subcloned to pminiMAD using KpnI/BamHI and the resulting plasmid (pTE87) was used for *clpP2* deletion.

For *clpE* deletion, fragments up- and downstream to *clpE* were amplified in PCRs using the oligonucleotides TE392/TE393 and TE394/TE391, respectively. The two fragments were joined by SOE-PCR and the resulting Δ*clpE* fragment was then cloned into pMAD by RF cloning (pTE99). The Δ*clpE* fragment of this plasmid was then subcloned into pminiMAD using KpnI/SalI, yielding the final pTE103 plasmid.

For deletion of *clpX*, fragments up- and downstream of *clpX* were first amplified using the primer pairs TE387/TE390 and TE388/TE389, respectively, then fused together in a SOE-PCR and cloned into pMAD by RF cloning (pTE97). The Δ*clpX* fragment of this plasmid was then subcloned into pHoss1 using KpnI/SalI, yielding the final pTE111 plasmid.

Plasmid pTE116 was generated for *yjbH* deletion. To this end, fragments up- and downstream of *yjbH* were amplified by PCR using TE411/TE412 and TE413/TE410 as the primers, respectively. Both fragments were then spliced together by SOE-PCR and cloned in pMAD using EcoRI/SalI as the restriction endonucleases.

For generation of plasmid pTE118, designed to facilitate *spxA1* deletion, fragments up- and downstream to *spxA1* were generated by PCR using oligonucleotides TE417/TE418 and TE419/TE416 as the primers. The resulting fragments were then spliced together in a SOE-PCR and cloned into pMAD via EcoRI/SalI, yielding plasmid pTE117. The Δ*spxA1* fragment of plasmid pTE117 was then subcloned into pminiMad using NcoI/SalI, which yielded the final *spxA1* deletion plasmid pTE118.

Plasmid pTE115 was constructed to facilitate IPTG-inducible *spxA1* expression in *L. monocytogenes*. This plasmid was obtained by cloning of a *spxA1* fragment, which was amplified by PCR from genomic DNA using the primer pair TE452/TE453, into pIMK3 using NcoI/SalI restriction.

Plasmid pPR5 was generated for IPTG-inducible *clpP2* expression in *L. monocytogenes*. For this, the *clpP2* open reading frame was amplified with primers PR19/PR20 and then ligated to pIMK3 using BamHI/SalI.

To facilitate immunologic detection of TimA, a Strep-tag was introduced at the C-terminus of *timA* present on plasmid pTE61 by PCR using TE379/TE380 as the primers. This yielded plasmid pTE107.

Plasmid pSW128 was generated for deletion of *qoxAB*. To this end, fragments up- and downstream of *qoxAB* were amplified using the oligonucleotides SW264/SW265 and SW268/SW269, respectively, fused together by SOE-PCR and cloned into pMAD using BamHI/SalI.

Plasmid pSW129 was generated for deletion of *cydAB*. Fragments up- and downstream of *cydAB* were amplified from chromosomal DNA using TE475/TE482 and TE480/TE481, respectively, as the primers. Both fragments were spliced together by SOE-PCR and then cloned into pHoss1 using XmaI/SalI.

Derivatives of pIMK3 were introduced into *L. monocytogenes* strains by electroporation and transformants were selected on BHI agar plates containing kanamycin at 37°C. Plasmid integration at the tRNA^Arg^ *attB* site was confirmed by PCR. Likewise, plasmid derivatives of pMAD, pminiMAD and pHoss1 were introduced into *L. monocytogenes*, but transformants were selected on BHI agar plates containing X-Gal and erythromycin at 30°C. The plasmid integration-excision protocols described by Arnaud *et al.* [56], Patrick and Kearns [57] and Abdelhamed *et al.* [55], respectively, were then used for gene deletions (and IPTG was added where necessary). All gene deletions were confirmed by PCR.

### Genome sequencing

Chromosomal DNA was isolated using the GenElute Bacterial Genomic DNA Kit (Sigma-Aldrich). 1 ng DNA and the Nextera XT DNA Library Prep Kit (Illumina) were used for library generation. Sequencing was carried out on MiSeq and NextSeq sequencers in paired-end sequencing modes of 2 x 300 bp and 2 x 150 bp, respectively. Reads were mapped to the *L. monocytogenes* EGD-e genome (NC_003210.1) [58] as the reference in Geneious (Biomatters Ltd.) and the alignment was analyzed using the Geneious SNP finder tool. Genome sequences of tartrolon resistant suppressor strains and the Δ*clpP2* mutant were deposited at the European Nucleotide Archive (ENA, https://www.ebi.ac.uk/) under project accession number PRJEB71775.

### Transposon insertion sequencing

An overnight culture of a previously constructed transposon insertion mutant library [27] was diluted to an OD_600_ of 0.1 and grown at 37°C for 5 h in BHI broth ± 0.25 µg/ml tartrolon B. 4 ml of this culture was harvested by centrifugation and resuspended in 400 µl buffer W (100 mM Tris-HCl, pH 8.0; 150 mM NaCl). 100 µl RNAse A buffer (QIAGEN) and 40 µl lysozyme (100 mg/ml) were added and the suspension incubated at 37°C overnight. The next day, chromosomal DNA was purified using phenol/choroform extraction followed by DNA precipitation using ethanol. The collected DNA pellet was resolved in 50 µl 5 mM Tris-HCl (pH 7.6).

The DNA concentration of each sample was determined using the NanoPhotometer (IMPLEN). 6 µg of chromosomal DNA was digested in a total volume of 200 µl using MmeI (NEB) according to the manufacturer´s instructions. Digestion was performed overnight, and the next day the reaction was inactivated at 65°C for 20 min. Dephosphorylation of the DNA fragments were done using 1.5 µl Quick CIP (NEB) according to manufacturer´s instructions. The dephosphorylated fragments were extracted using the NucleoSpin Gel and PCR clean-up kit (Macherey-Nagel) according to the manufacturer´s instructions. The elution step was performed using 30 µl ddH_2_O.

Adapters MF38 and MF52, needed for subsequent sequencing were mixed equimolarly to a final concentration of 50 µM and incubated for 10 min at 95°C, and cooled down at room temperature for 15 minutes. Adapters were ligated with the eluted DNA using the T4 DNA ligase according to manufacturer´s instructions at 6°C overnight. The next day, a clean-up was performed using the Size selection kit (QIAGEN) according to manufacturer´s instructions.

Eluted fragments were used as a template in a PCR containing 10 pM of primers MF38/MF53. Fragment amplification was done using the Phusion high fidelity polymerase (NEB) according to manufacturer´s instructions. Products with the desired size of 165 base pairs were extracted from the reaction mix by agarose gel electrophoresis using 1% (w/v) agarose and purified with the NucleoSpin Gel and PCR clean-up kit (Macherey-Nagel).

The adapter-linked PCR products were used as a template to introduce multiplex identifiers for pooled sequencing. PCR was performed with the following conditions: 2.5 μL template (3 ng/μL), 2.5 μL Nextera XT index primer (each N7 and S5, Illumina), and 12.5 μL 2x KAPA HiFi HotStart ReadyMix (Roche) and 5 μL H_2_O using a PCR program with these settings: 3 min at 95°C; eight cycles (each 30 sec) of 5 min at 95°C, 55°C, 72°C,72°C; and storage at 4°C until bead-clean up. Sequencing was performed in a 2×150-bp paired-end run on an Illumina NextSeq 2000 with P2 chemistry.

### Bioinformatic processing of Tn-Seq sequencing reads and prediction of genes required for tartrolon B resistance

Sequencing raw reads were processed using a semi-automated pipeline on the RKI Linux Server 07 (Debian GNU/Linux 9.13). Forward and reverse reads were trimmed to remove sequencing primers and transposon sequence using cutadapt (version 1.12), resulting in 13 bp fragments matching the flanking region of the integration side of the transposon. Fragments were mapped on the closed EGD-e genome (NC_003210.1) [58] allowing zero mismatch using bowtie (version 1.1.2) [59]. To remove sequencing imbalance between samples, mapped reads were reduced to 2 x 10^6^ reads per sample. These read files were deposited at ENA under project accession number PRJEB71775. Further analysis to identify conditionally essential genes was conducted using TnSeq Explorer (version 1.5b) [60] using the NCBI Gene Bank file with the ORF locations and annotation. To identify essential genes under the respective experimental conditions, mapping window size was set to 200 bp. Transposon insertion in the initial 5% of the 5’ region and the last 20% of the 3’ region of the ORF were ignored. A gene was defined as required in the presence of tartrolon B, if the insertion density was reduced at least two-fold with a *p*<0.01 (*t*-*t*est, n=3) after tartrolon B exposure.

### Determination of minimal inhibitory concentrations

For MIC determination, an overnight culture was diluted to an OD_600_ of 0.1 in BHI medium. A Cellstar 96 well cell culture plate with flat bottom (Greiner Bio-One) was loaded with 100 µl BHI medium containing double the concentration of the antibiotic to be tested. 100 µl of the diluted overnight culture was added to each well. Growth curves were performed in a Multiskan Sky or Multiskan Go Microplate Spectrophotometer (Thermo Fisher Scientific) at 37°C with 5 seconds shaking followed by a 5 second break for 24 hours. The OD_600_ was measured every 5 minutes. The MIC was defined as the lowest concentration where no bacterial growth could be observed after 20 hours.

### Membrane activity profiling of tartrolon B

*L. monocytogenes* strain EGD-e was grown in BHI broth at 37°C to exponential growth phase (OD_600_ ~0.8-1.0), pelleted by centrifugation and resuspended in 1 ml PBS. DiSC_3_(5) or TO-PRO-3 iodide were added to parallel samples at a final concentrations of 1 µM each. Stained cells were incubated at room temperature for 20 min and fluorescence emission was recorded using a Tecan Infinite M1000 fluorescence microplate reader using λ_ex_=610 nm and λ_em_=660 nm as the excitation and emission wavelengths, respectively, prior and immediately subsequent to addition of nisin (final concentration: 1 mg/ml) and tartrolon B (1 µg/ml).

### Isolation of cellular proteins, SDS-PAGE and Western blotting

In order to isolate cellular proteins from *L. monocytogenes* strains, cells from a 20 ml culture volume were harvested by centrifugation and washed once with ZAP buffer (10 mM Tris/HCl pH7.5, 200 mM NaCl). Thereafter, the pellet was resuspended in 1 ml ZAP buffer + 1 mM PMSF and cells were disrupted by sonication. Debris was removed by centrifugation and the supernatant, representing the total cellular protein fraction, was used for further analysis. Aliquots were separated by SDS polyacrylamide gel electrophoresis (PAGE) and stained using a colloidal Coomassie stain or transferred onto positively charged polyvinylidene fluoride membranes using a semi-dry transfer unit. DivIVA, MurA and SpxA1 were stained using polyclonal rabbit antisera recognizing *B. subtilis* MurAA [26,61], DivIVA [62,63] or Spx (received from Ulf Gerth, University of Greifswald, Germany) as the primary antibodies, respectively. An anti-rabbit immunoglobulin G conjugated to horseradish peroxidase (HRP) was used as the secondary antibody. Strep-tagged proteins were detected using an anti-Strep antibody conjugated to HRP (IBA-Lifesciences, Göttingen, Germany). HRP was detected on the membranes in a chemiluminescence imager (Chemidoc Imaging System, Bio-rad) using the ECL chemiluminescence detection system (Thermo Scientific).

### Protein purification

ClpP2-Strep proteins (carrying C-terminal Strep-tag II sequences) were overproduced in *E. coli* BL21(DE3). To this end, 500 ml LB broth containing 100 µg/ml ampicillin were inoculated with *E. coli* BL21 cells carrying the corresponding vector construct to an initial OD_600_ of 0.1. Cells were grown at 37 °C and 250 rpm and 1 mM IPTG was added when an OD_600_ of 0.6 – 0.8 was reached. After three more hours, cells were pelleted by centrifugation (8,000 × *g*, 5 min, 4°C) and the pellet was washed once with 25 ml buffer W (100 mM Tris-HCl pH 8.0; 150 mM NaCl). Cells were resuspended in 40 ml buffer W and lysed using an Emulsiflex homogenizer (Avestin, Germany). Cell debris was removed by centrifugation (8,000 × *g*, 20 min, 4°C), and the resulting supernatant was filtered through a Minisart filter with a pore size of 0.45 μm (Sartorius). The strep tagged proteins were purified using affinity chromatography with Strep-Tactin Sepharose (IBA Lifesciences, Germany) according to the manufacturer’s instructions. Fractions containing purified proteins were pooled, aliquoted, and stored at -20°C.

### ClpP2 peptidase assay

Peptidase reactions were carried out with 20 µM (monomeric) of ClpP2-Strep or ClpP2 E9K-Strep proteins in reaction buffer (100 mM Tris, pH 8.0, 200 mM NaCl) and a final volume of 100 µl and various concentrations of ADEP2 and tartrolon B (final concentrations: 0 µM, 1 µM, 2 µM, 4 µM). ADEP2 and tartrolon B stock solutions were generated with DMSO as the solvent. The reaction was started by addition of 10 µl of 2 mM Suc-Leu-Tyr-7-amido-4-methylcoumarin and fluorescence emission was monitored in black flat bottom 96 well plates (Thermo Scientific Nunc MicroWell) at 32 °C in a Tecan infinite M1000 reader with excitation at 380 nm and emission at 440 nm. Assays were conducted for 3 h with 5 minutes between each measurement step.

### J774 macrophage infection

Intracellular growth of *L. monocytogenes* strains was determined using J774.A1 mouse macrophages. These experiments were performed as described earlier [64].

## Supporting information

S1 Fig Absence of *clpP1* does not affect tartrolon B resistance of *L. monocytogenes*. Growth of *L. monocytogenes* strains EGD-e (wt, left panel) and LMPR6 (Δ*clpP1*, right panel) in BHI broth at 37°C containing increasing concentrations of tartrolon B.
(TIF)

S2 Fig Resistance of the Δ*clpP2* mutant against aurantimycin A, boromycin and nigericin. Minimal inhibitory concentrations of aurantimycin A, boromycin, nigericin and tartrolon B (for comparison) for *L. monocytogenes* strains EGD-e (wt) and LMTE80 (Δ*clpP2*). The experiment was repeated three to four times and average values and standard deviations were calculated. The asterisk marks statistically significant differences (** - *P*<0.01, * - P<0.05, *t*-tes*t*). n. s. – not significant.
(TIF)

S3 Fig Uncropped Western blot images for the experiments shown in Fig 2B (left panel) and Fig 5D (right panel) in the main manuscript.
(TIF)

S4 Fig Growth of *L. monocytogenes clpP2* mutants in BHI broth and inside macrophages. (A) Growth of *L. monocytogenes* strains EGD-e (wt), LMTE38 (*clpP2 E9K*), LMTE74 (*clpP2 T90I*) and LMTE80 (Δ*clpP2*) in BHI broth at 37°C (left panel) and 42°C (right panel). (B) Complementation of the Δ*clpP2* growth defect. Growth of *L. monocytogenes* strains EGD-e (wt), LMTE80 (Δ*clpP2*) and LMTE90 (i*clpP2*) in BHI broth ± 1 mM IPTG at 42°C. Average values and standard deviations from experiments performed in triplicate are shown.(C) Intracellular growth of the same set of strains in J774 mouse macrophages. The experiment was carried out in triplicate and average values and standard deviations were calculated. Asterisks mark statistically significant differences compared to the wild type (*P*<0.01, *t*-*t*est with Bonferroni-Holm correction). Strain LMS250 (Δ*hly*) was included as negative control.
(TIF)

S5 Fig Purity of *L. monocytogenes* ClpP2-Strep and ClpP2 E9K-Strep samples. Aliquots of both proteins were separated by SDS-PAGE and stained using a colloidal Coomassie stain.
(TIF)

S6 Fig Effect of SpxA1 depletion on growth of *L. monocytogenes*. Growth of *L. monocytogenes* strains EGD-e (wt) and LMTE139 (i*spxA1*) in BHI broth ± 1 mM IPTG at 37°C. Average values and standard deviations, which were calculated from technical triplicates, are shown.
(TIF)

S7 Fig Uncropped Western blot images for the experiment shown in Fig 7D in the main manuscript.
(TIF)

S8 Fig Effect of a *yjbH* deletion on P_*timABR*_ promoter activity. P_*timABR*_*-lacZ* mediated β-galactosidase activity in *L. monocytogenes* strains LMTE19 (wt), LMTE147 (Δ*yjbH*), LMTE50 (Δ*timR*) and LMTE142 (Δ*timR* Δ*yjbH*) during exponential growth in BHI broth containing different tartrolon B concentrations. Strains LMSH16 (*lacZ*) and LMSH98 (P_*lieAB*_*-lacZ* Δ*lftR*) were included as negative and positive controls, respectively. The experiment was carried out three times and average values and standard deviations are shown.
(TIF)

S9 Fig Checkerboard assay to test for a possible synergism of tartrolon B and hydrogen peroxide. *L. monocytogenes* strain EGD-e was grown at 37°C in a microtiter plate containing BHI broth and different combinations of increasing concentrations of tartrolon B and hydrogen peroxide. Growth was recorded in a plate reader and the obtained growth curves for each combination are shown. Hydrogen peroxide did not sensitize *L. monocytogenes* against tartrolon B and *vice versa*, thus, combined tartrolon B and hydrogen peroxide treatment does not generate a synergistic effect in the prevention of *L. monocytogenes* growth.
(TIF)

S1 Table Effect of potassium on tartrolon resistance of SpxA1-depleted *L. monocytogenes* cells. Minimal inhibitory concentrations of tartrolon B for *L. monocytogenes* strains EGD-e (wt) and LMTE139 (i*spxA1*) grown in BHI broth ± 1 mM IPTG in the absence and presence of 250 mM potassium chloride.
(XLSX)

S2 Table Raw data for the results shown in Table 1 (Genes required for tartrolon B resistance according to Tn-Seq).
(XLSX)

S1 Data Underlying numerical data for all of our graphs and summary statistics.
(7Z)

S1 Striking image caption Genetic network for tartrolon B resistance of *Listeria monocytogenes*. Genes for compound sensing, compound export and attenuation of toxic compound effects cooperate in resistance development.
(TIF)
