# Supplementary figures and images for: ClpP2 proteasomes and SpxA1 determine Listeria monocytogenes tartrolon B hyper-resistance
Source: PLoS Genet. 2025 Apr 4;21(4):e1011621. doi: 10.1371/journal.pgen.1011621 (PMC11970672; doi:10.1371/journal.pgen.1011621)

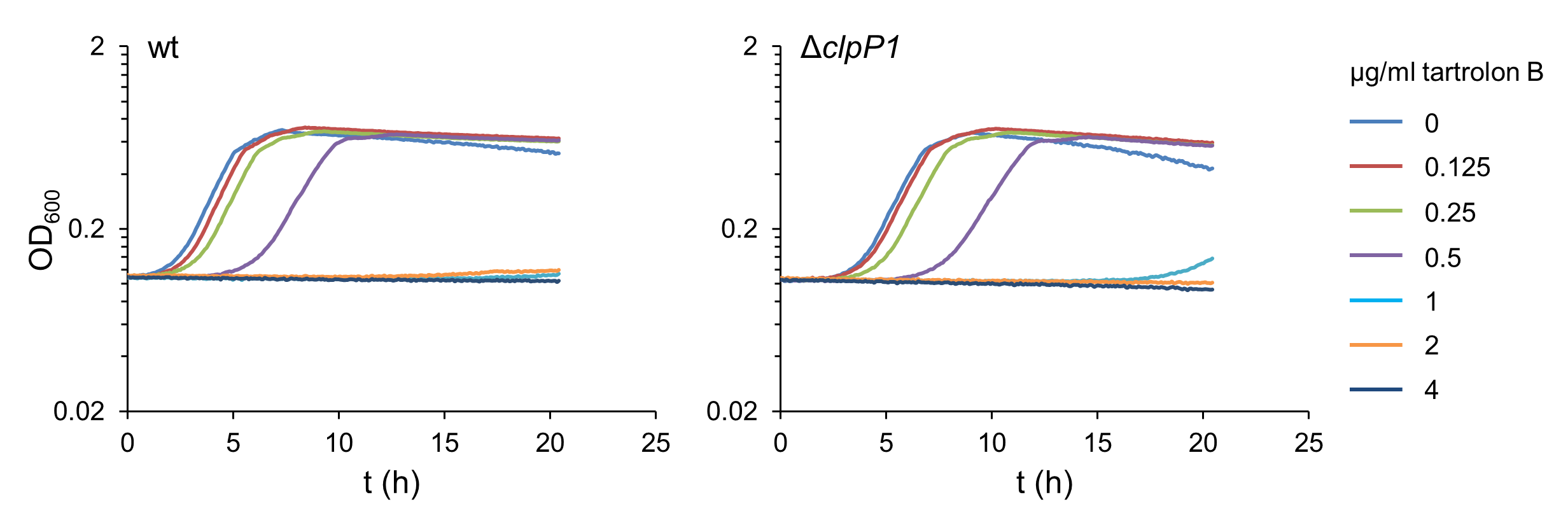

Supplement: S1 Fig — Absence of clpP1 does not affect tartrolon B resistance of L. monocytogenes. Growth of L. monocytogenes strains EGD-e (wt, left panel) and LMPR6 (ΔclpP1, right panel) in BHI broth at 37°C containing increasing concentrations of tartrolon B. (TIF) [file pgen.1011621.s001.TIF]

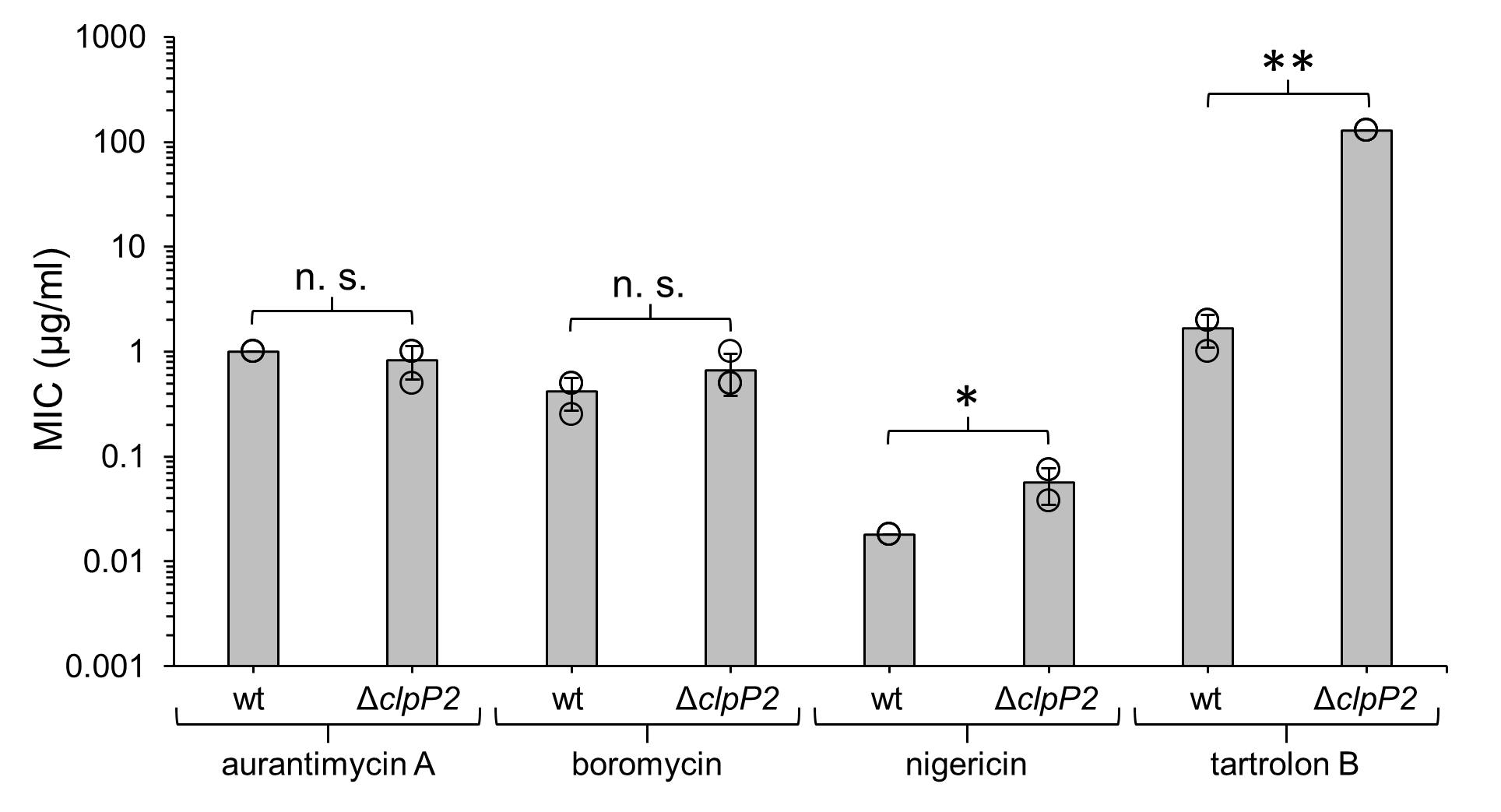

Supplement: S2 Fig — Resistance of the ΔclpP2 mutant against aurantimycin A, boromycin and nigericin. Minimal inhibitory concentrations of aurantimycin A, boromycin, nigericin and tartrolon B (for comparison) for L. monocytogenes strains EGD-e (wt) and LMTE80 (ΔclpP2). The experiment was repeated three to four times and average values and standard deviations were calculated. The asterisk marks statistically significant differences (** - P<0.01, * - P<0.05, t-test). n. s. – not significant. (TIF) [file pgen.1011621.s002.TIF]

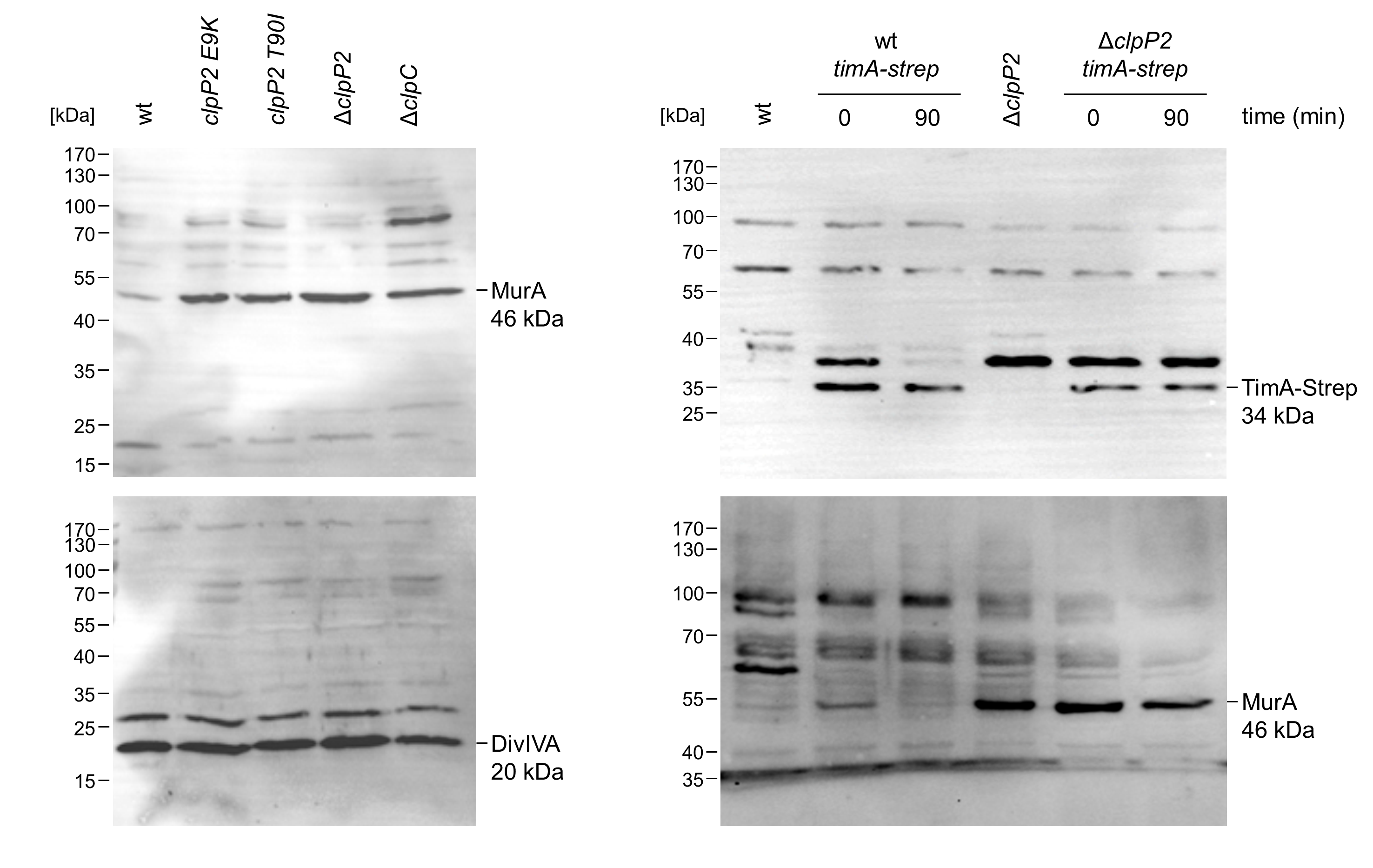

Supplement: S3 Fig — Uncropped Western blot images for the experiments shown in Fig 2B (left panel) and Fig 5D (right panel) in the main manuscript. (TIF) [file pgen.1011621.s003.TIF]

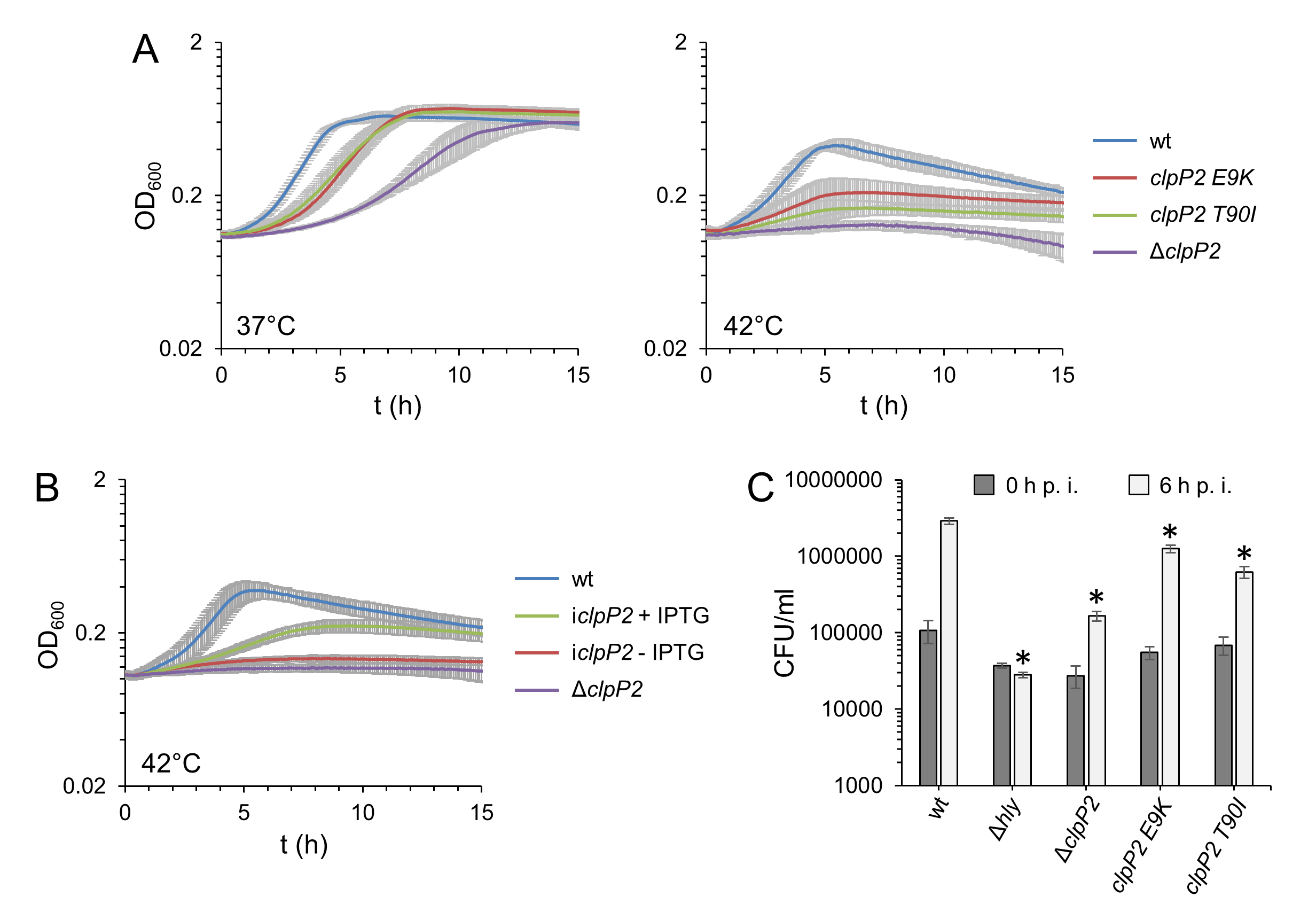

Supplement: S4 Fig — Growth of L. monocytogenes clpP2 mutants in BHI broth and inside macrophages. (A) Growth of L. monocytogenes strains EGD-e (wt), LMTE38 (clpP2 E9K), LMTE74 (clpP2 T90I) and LMTE80 (ΔclpP2) in BHI broth at 37°C (left panel) and 42°C (right panel). (B) Complementation of the ΔclpP2 growth defect. Growth of L. monocytogenes strains EGD-e (wt), LMTE80 (ΔclpP2) and LMTE90 (iclpP2) in BHI broth ± 1 mM IPTG at 42°C. Average values and standard deviations from experiments performed in triplicate are shown.(C) Intracellular growth of the same set of strains in J774 mouse macrophages. The experiment was carried out in triplicate and average values and standard deviations were calculated. Asterisks mark statistically significant differences compared to the wild type (P<0.01, t-test with Bonferroni-Holm correction). Strain LMS250 (Δhly) was included as negative control. (TIF) [file pgen.1011621.s004.TIF]

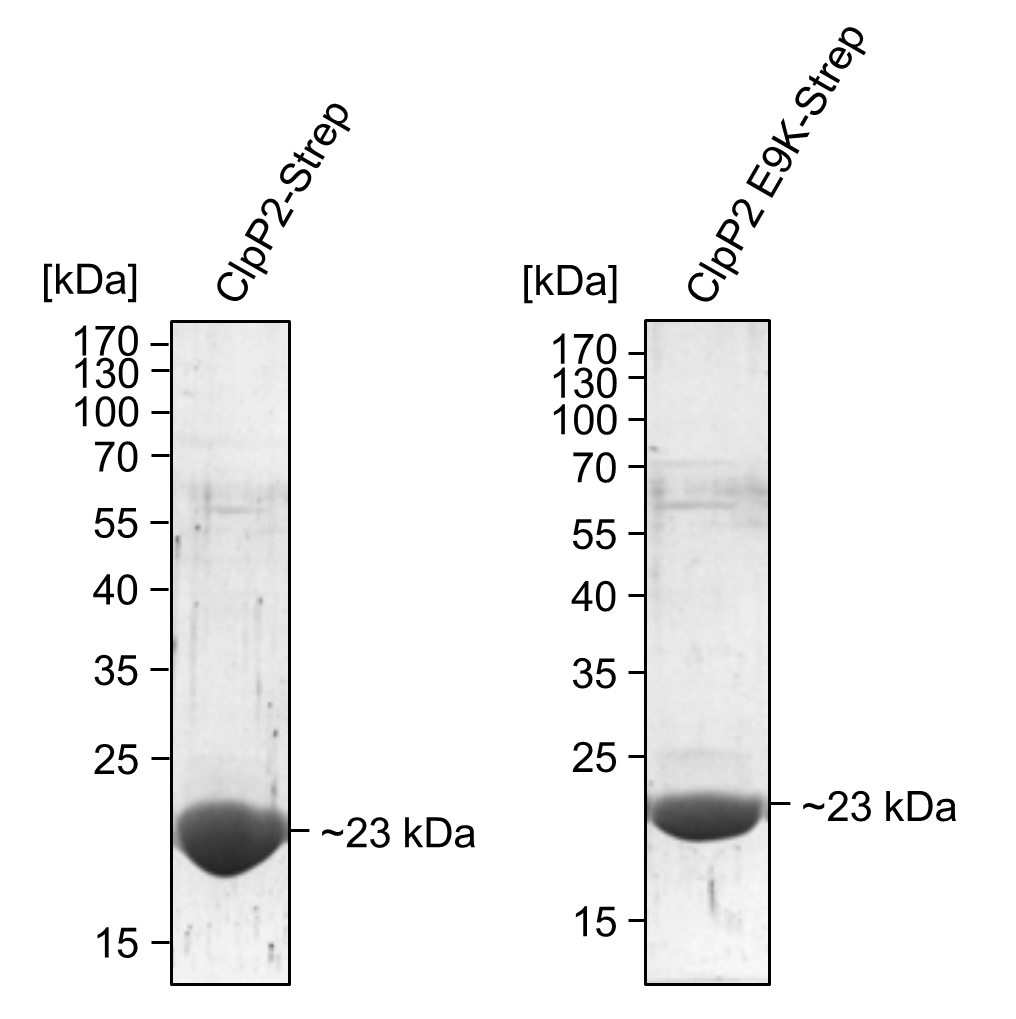

Supplement: S5 Fig — Purity of L. monocytogenes ClpP2-Strep and ClpP2 E9K-Strep samples. Aliquots of both proteins were separated by SDS-PAGE and stained using a colloidal Coomassie stain. (TIF) [file pgen.1011621.s005.TIF]

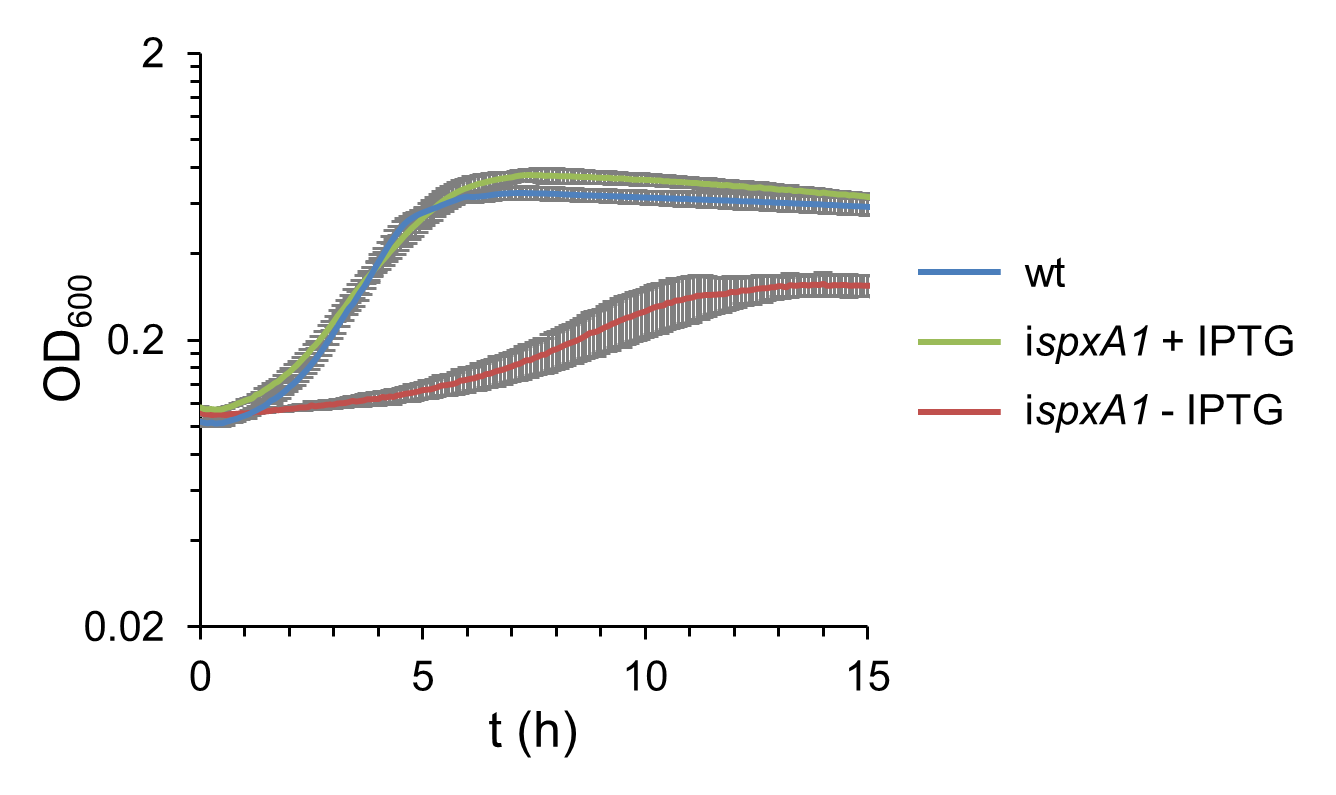

Supplement: S6 Fig — Effect of SpxA1 depletion on growth of L. monocytogenes. Growth of L. monocytogenes strains EGD-e (wt) and LMTE139 (ispxA1) in BHI broth ± 1 mM IPTG at 37°C. Average values and standard deviations, which were calculated from technical triplicates, are shown. (TIF) [file pgen.1011621.s006.TIF]

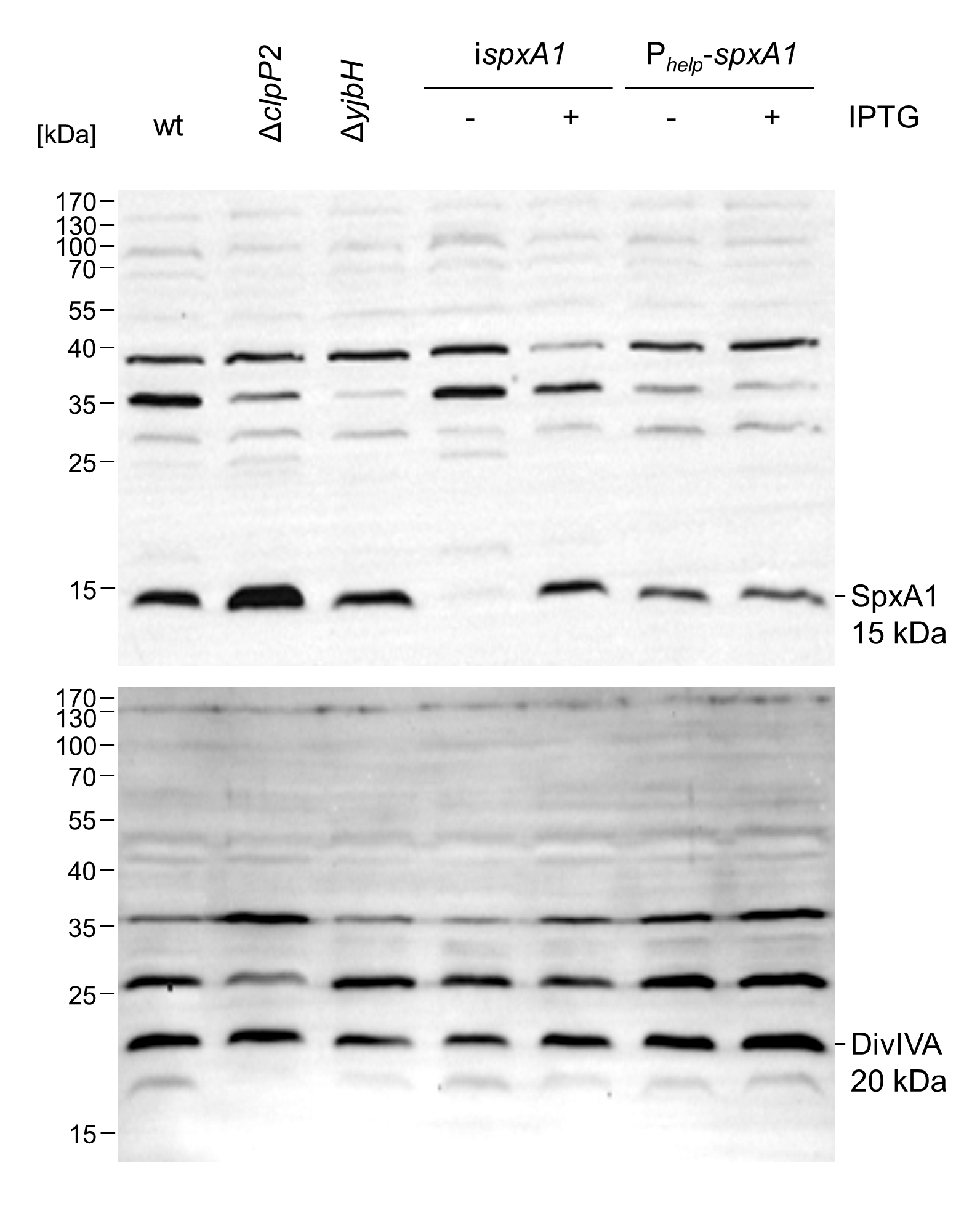

Supplement: S7 Fig — Uncropped Western blot images for the experiment shown in Fig 7D in the main manuscript. (TIF) [file pgen.1011621.s007.TIF]

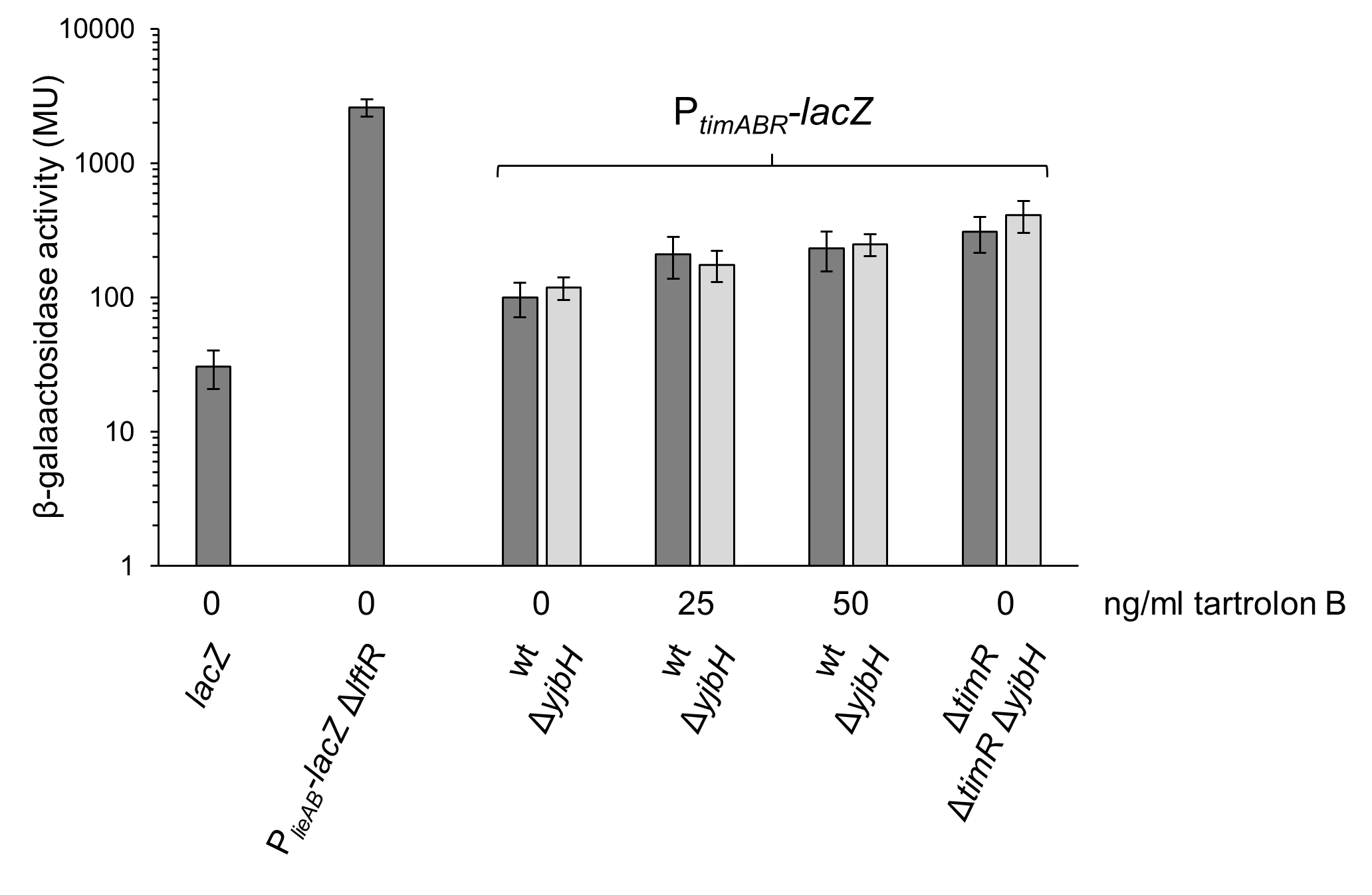

Supplement: S8 Fig — Effect of a yjbH deletion on PtimABR promoter activity. PtimABR-lacZ mediated β-galactosidase activity in L. monocytogenes strains LMTE19 (wt), LMTE147 (ΔyjbH), LMTE50 (ΔtimR) and LMTE142 (ΔtimR ΔyjbH) during exponential growth in BHI broth containing different tartrolon B concentrations. Strains LMSH16 (lacZ) and LMSH98 (PlieAB-lacZ ΔlftR) were included as negative and positive controls, respectively. The experiment was carried out three times and average values and standard deviations are shown. (TIF) [file pgen.1011621.s008.TIF]

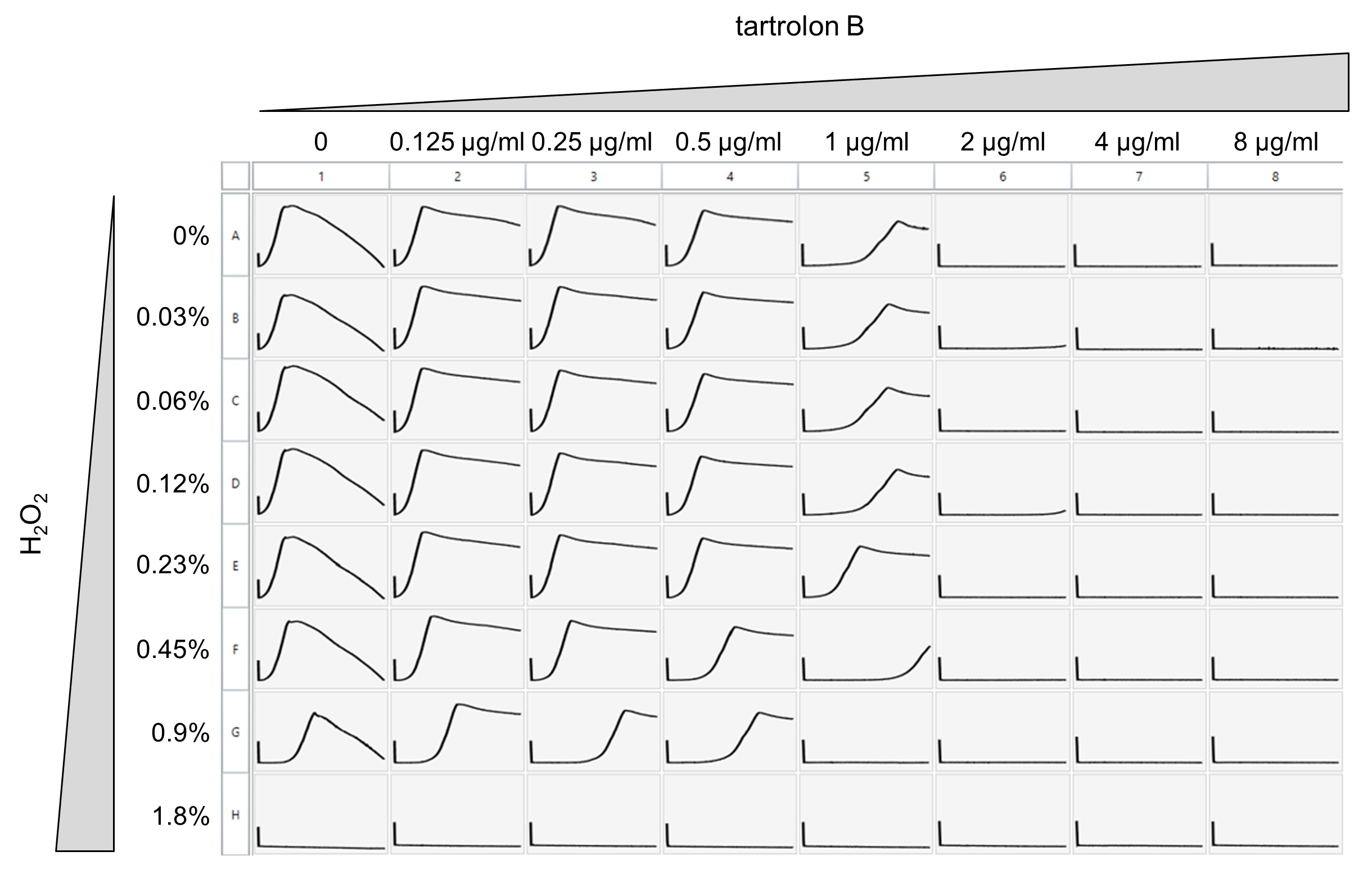

Supplement: S9 Fig — Checkerboard assay to test for a possible synergism of tartrolon B and hydrogen peroxide. L. monocytogenes strain EGD-e was grown at 37°C in a microtiter plate containing BHI broth and different combinations of increasing concentrations of tartrolon B and hydrogen peroxide. Growth was recorded in a plate reader and the obtained growth curves for each combination are shown. Hydrogen peroxide did not sensitize L. monocytogenes against tartrolon B and vice versa, thus, combined tartrolon B and hydrogen peroxide treatment does not generate a synergistic effect in the prevention of L. monocytogenes growth. (TIF) [file pgen.1011621.s009.TIF]

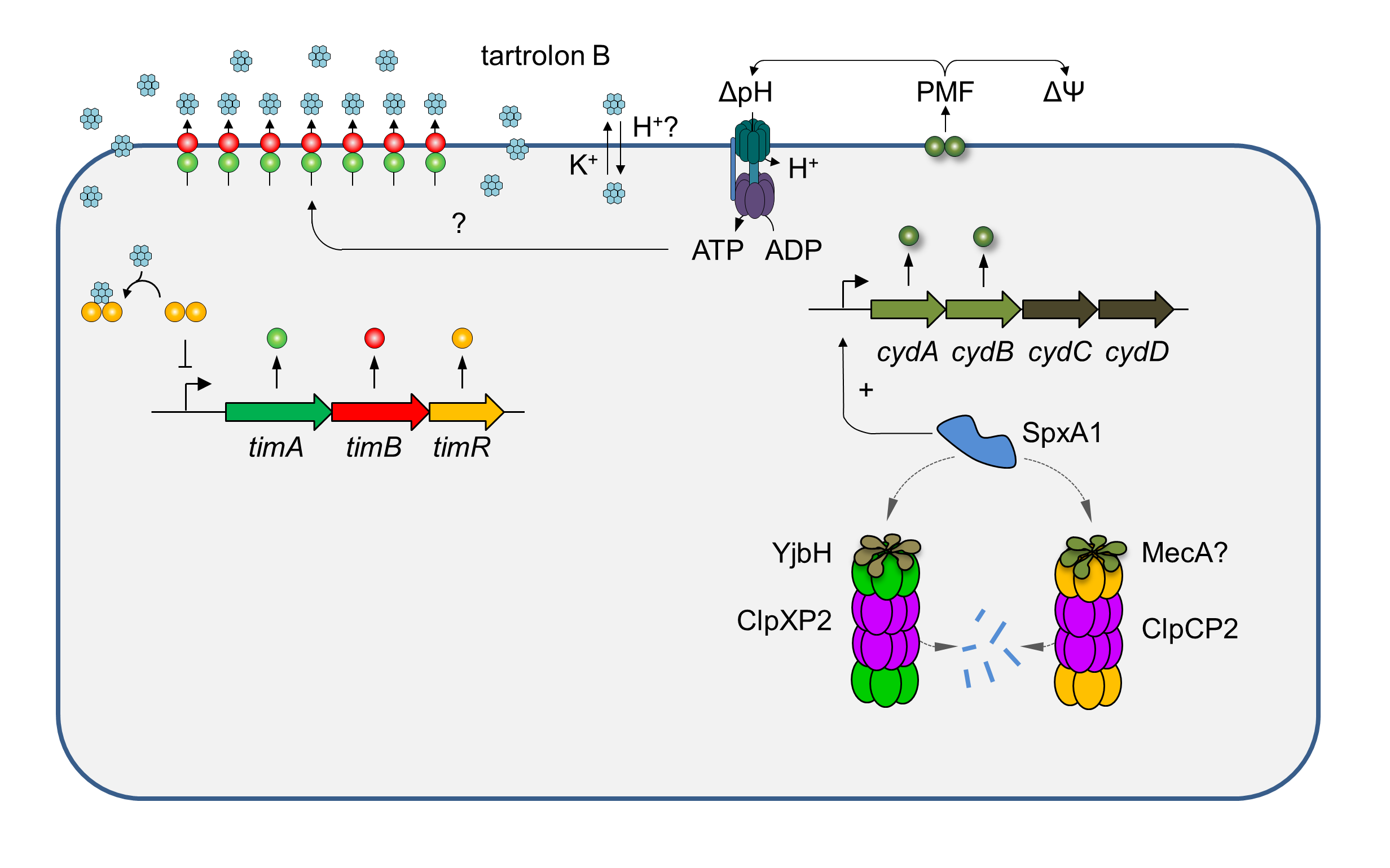

Supplement: S1 Striking image caption — Genetic network for tartrolon B resistance of Listeria monocytogenes. Genes for compound sensing, compound export and attenuation of toxic compound effects cooperate in resistance development. (TIF) [file pgen.1011621.s013.tif]
